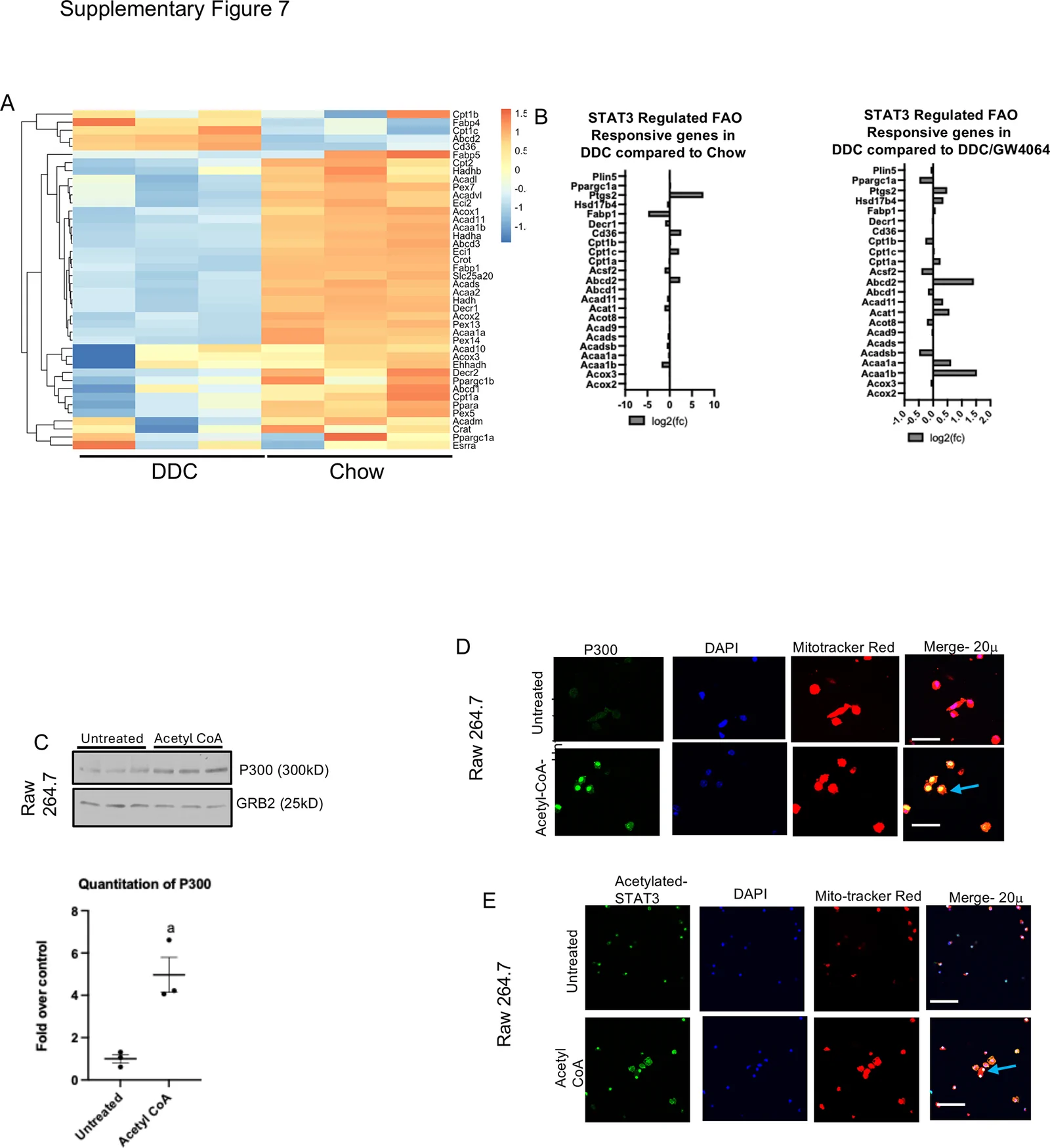# Farnesoid X receptor activates STAT3 to promote fatty acid oxidation and suppress inflammatory macrophage responses in cholestasis

**DOI:** 10.1016/j.jbc.2026.113508

**Published:** 2026-08-28

**Authors:** Swati Ghosh, Michael W. Devereaux, Colin C. Anderson, Amy Argabright, David J. Orlicky, Ronald J. Sokol

**Affiliations:** 1 Section of Pediatric Gastroenterology, Hepatology and Nutrition, Department of Pediatrics, University of Colorado School of Medicine, Aurora, Colorado, USA; 2 Department of Biochemistry and Molecular Genetics, University of Colorado School of Medicine, Aurora, Colorado, USA; 3 Department of Pathology, University of Colorado School of Medicine, Aurora, Colorado, USA; 4 Pediatric Liver Center, Digestive Health Institute, Children’s Hospital Colorado, Aurora, Colorado, USA

**Keywords:** cholestasis, primary sclerosing cholangitis, macrophage, biliary atresia, hepatocyte, cholangiocyte

## Abstract

Inflammatory cholangiopathies, including primary sclerosing cholangitis, involve bile duct injury, macrophage-driven inflammation, and progressive fibrosis. While farnesoid X receptor (FXR) regulates bile acid metabolism and immune responses, its role in macrophage immunometabolic reprogramming during cholestasis remains unclear. Here, we investigated whether FXR regulates liver immunometabolism through STAT3-dependent mechanisms in cholestatic liver disease. Using transcriptomic, oxylipin lipidomic, histological, and metabolomic analyses in a chronic murine model of DDC-induced cholangiopathy, we explored the role of FXR-induced STAT3 signaling in regulating inflammatory and metabolic responses. FXR agonists significantly reduced biochemical and histologic cholestatic liver injury as well as suppression of macrophage infiltration. Liver RNA-seq revealed that cholestatic injury activated inflammatory, TGF-β, and arachidonic acid metabolic pathways and reduced fatty acid oxidation (FAO) programs, while FXR restored pathways linked to FAO. Mechanistically, FXR activation in the liver increased STAT3 phosphorylation, acetylation and mitochondrial translocation, boosted STAT3 binding to promoters of genes involving FAO pathways, and triggered the mRNA expression of FAO genes. Pharmacologic STAT3 activation with colivelin reproduced protective effects of GW4064-mediated FXR activation. Conversely, co-administration of the STAT3 inhibitor C-188-9 partially reversed GW4064-mediated suppression of inflammatory and fibrogenic gene programs and reduced induction of FAO genes. Arachidonic acid injection of mice induced macrophage activation and hepatocyte apoptosis, both of which were attenuated by GW4064. STAT3 siRNA knockdown in hepatocyte and macrophage cell systems also blunted FXR agonist-induced transcriptional responses. These findings identify a novel FXR agonist–driven STAT3 immunometabolic axis that integrates bile acid signaling, FAO, and macrophage reprogramming to regulate inflammation during cholestatic liver injury.

Inflammatory cholangiopathies, including primary sclerosing cholangitis (PSC) and biliary atresia, are marked by bile duct injury, inflammation, and ongoing fibrosis that can lead to liver failure (1, 2). Despite progress in understanding bile acid biology and immune regulation in the liver, effective treatments for most cholestatic liver diseases are still limited. Increasing evidence shows that cholestatic injury is caused not only by bile acid toxicity but also by inflammatory signaling and immune cell activation within the liver. Specifically, hepatic macrophages act as important sensors of biliary damage and coordinate inflammatory responses that promote cholangiocyte activation, ductular reaction, and fibrogenesis (2, 3).

The nuclear receptor farnesoid X receptor (FXR) is a key regulator of bile acid balance and liver-related metabolism. FXR regulates bile acid production, transport, and detoxification, helping protect liver cells from damage caused by accumulated bile acids (4). In addition to its role in metabolism, FXR also has anti-inflammatory effects in the liver and intestines. FXR agonists reduce inflammation and enhance liver healing in experimental models of cholestasis (5, 6, 7, 8), leading to human clinical trials of FXR agonists as potential treatments for cholestatic liver diseases.

Signal transducer and activator of transcription 3 (STAT3) is a transcription factor that plays a vital role in hepatocyte survival, liver regeneration, and inflammatory regulation. STAT3 signaling is activated downstream of cytokines like interleukin-6 and governs transcriptional programs that control cell survival and immune responses in the liver (9, 10). Genetic studies show that hepatocyte-specific deletion of STAT3 increases vulnerability to liver injury, emphasizing its protective role in hepatic inflammation and tissue repair. Emerging evidence suggests that macrophage activation during liver injury is closely linked to metabolic reprogramming. Pro-inflammatory macrophages typically exhibit glycolytic metabolism, whereas anti-inflammatory macrophages rely on mitochondrial oxidative metabolism and fatty acid oxidation to support tissue repair and immune resolution (11). Nuclear receptors and metabolic signaling pathways have been proposed as important regulators of macrophage immunometabolism; however, the mechanisms linking bile acid signaling to macrophage immunometabolic reprogramming during cholestatic liver injury remain poorly understood.

Lipid mediator pathways also contribute to inflammatory signaling in liver disease. Arachidonic acid (AA)-derived eicosanoids, including prostaglandins and leukotrienes, regulate immune cell activation and cytokine production in inflammatory disorders (12, 13). Dysregulated AA metabolism has been implicated in metabolic inflammation and chronic liver disease, but its role in macrophage activation in cholangiopathies remains unclear. Many AA, fatty acid oxidation (FAO), and TCA metabolites also exhibit anti-inflammatory effects (14).

While FXR agonists have been shown to protect against cholestatic liver injury and STAT3 signaling has been implicated in hepatoprotection, it remains unknown how FXR-induced STAT3 signaling regulates macrophage immunometabolism during cholangiopathy. In particular, the relationship between FXR-STAT3 signaling, fatty acid oxidation, AA-derived lipid mediators, and macrophage polarization has not been defined. Addressing this gap is important because macrophage metabolic reprogramming is increasingly recognized as a key determinant of inflammation resolution and tissue repair in chronic liver disease.

Based on these observations, we hypothesized that FXR signaling regulates macrophage immunometabolism in cholestatic injury through STAT3-dependent mechanisms that link lipid mediator signaling with metabolic reprogramming. Using transcriptomic, metabolomic, and functional analyses in a mouse model of cholangiopathy, we show that FXR activation suppresses AA-driven pro-inflammatory signaling, promotes STAT3-dependent fatty acid oxidation, and shifts hepatic macrophages toward an anti-inflammatory phenotype. These changes ultimately reduce cholangiocyte injury, inflammatory signaling, and fibrotic remodeling. Together, our findings reveal a previously unrecognized FXR–STAT3 immunometabolic axis that connects bile acid signaling, lipid mediator metabolism, and macrophage function during cholestatic liver injury, suggesting potential therapeutic strategies targeting macrophage immunometabolism in cholestatic disorders.

## Results

### FXR agonism attenuates DDC-induced cholestatic injury and reprograms hepatic inflammatory and metabolic gene expression

To elucidate the role of FXR in a murine model of inflammatory cholangiopathy, we administered the 0.1% DDC diet to adult male C57/BL6 mice (DDC mice) for 14 days. In subgroups, either the FXR agonist GW4064 (30 mg/kg body weight per day) or obeticholic acid (OCA; 20 mg/kg body weight per day) were administered intravenously (i.v.) in DDC mice for 14 days through a central venous catheter. DDC mice exhibited severe hepatobiliary injury, as evidenced by significant elevations in serum AST, ALT, alkaline phosphatase, total bilirubin, and total bile acids compared with Chow-fed controls (Fig. 1*A*). Pharmacological activation of FXR significantly prevented cholestatic injury. OCA, and to a lesser extent GW4064, significantly reduced serum total bilirubin, alkaline phosphatase, and total serum bile acids compared to DDC-treated mice. AST and ALT were more modestly improved in OCA and GW4064-treated mice. Histology of the liver further supported the protective effects of FXR activation. Hematoxylin and eosin (H&E) staining demonstrated prominent portal inflammation, hepatocellular injury, and ductular reaction in DDC mice, whereas treatment with GW4064 or OCA substantially attenuated these pathological features (Fig. 1*B*). Immunohistochemical staining for the macrophage marker F4/80 revealed pronounced periportal macrophage accumulation in DDC livers that was significantly diminished following treatment with both FXR agonists. Similarly, CK19 staining showed a prominent ductular reaction in DDC-treated mice that was markedly reduced in GW4064 and OCA-treated groups. Quantitative analysis confirmed significant decreases in both F4/80-positive macrophage infiltration and CK19-positive ductular structures following FXR activation.

**Figure 1:**
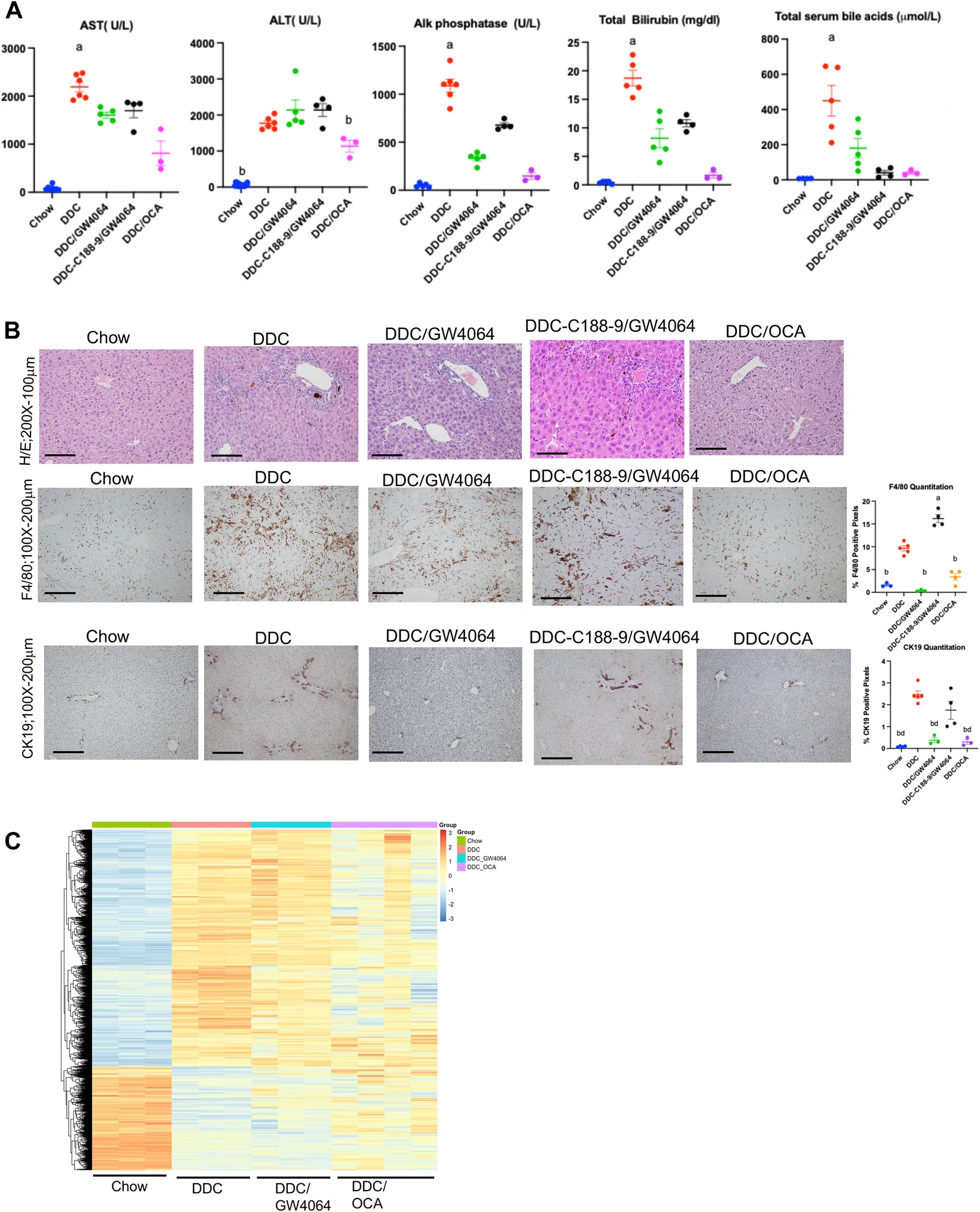

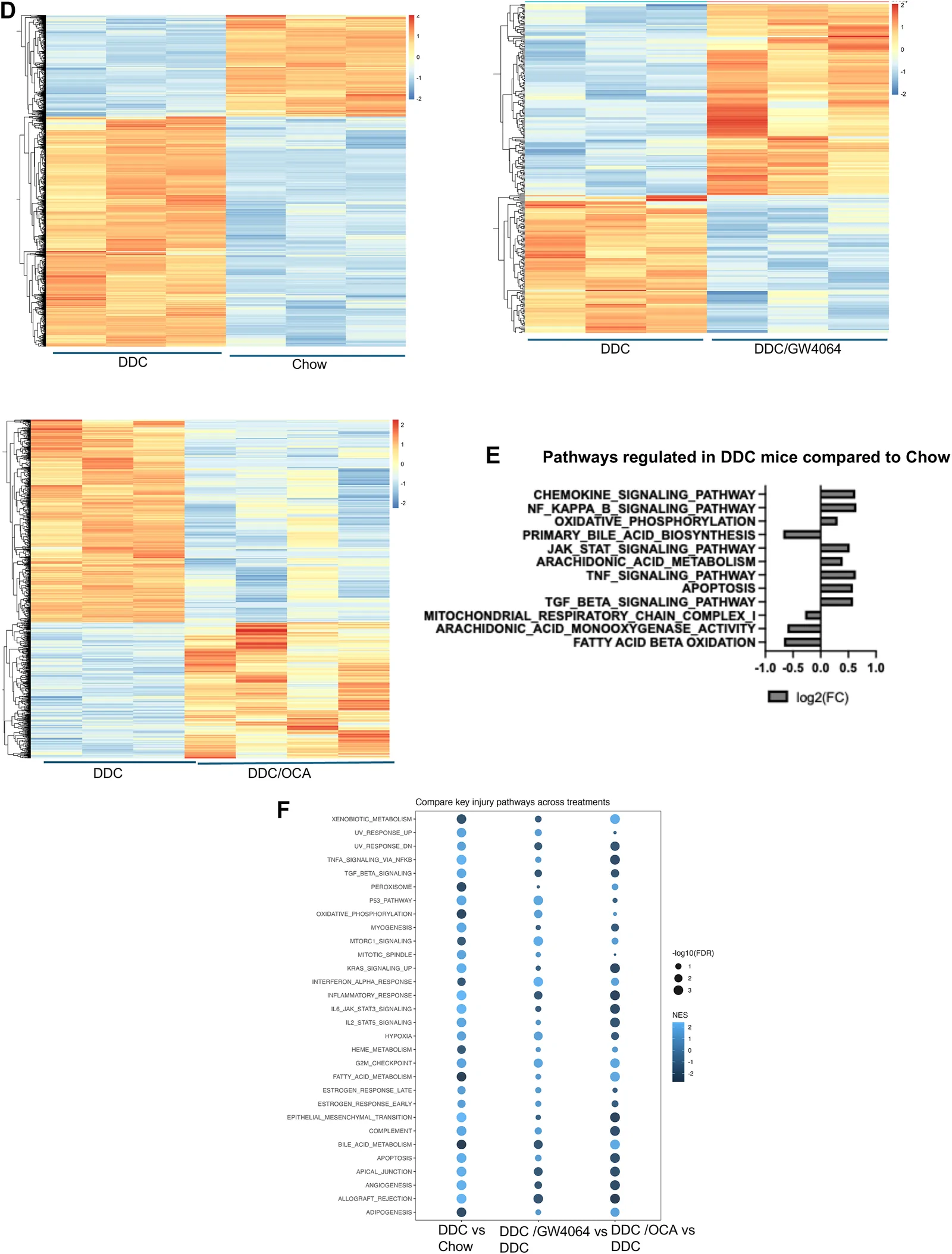
**FXR agonists attenuate DDC-induced cholestatic liver injury and reprogram hepatic transcriptional responses.***A*, Serum biochemical markers of liver injury and cholestasis in mice fed Chow or DDC diet with or without treatment of FXR agonists GW4064 or OCA, or the STAT3 inhibitor, C-188-9. Serum AST, ALT, alkaline phosphatase, total bilirubin, and total bile acids were significantly higher in 14-days DDC-fed mice compared to Chow controls and were significantly decreased after FXR agonist treatment. Co-administration of the STAT3 inhibitor C-188-9 in DDC mice partially attenuated (alkaline phosphatase and total bilirubin) the protective effects of GW4064. *B*, representative liver histology and immunohistochemistry. Hematoxylin and eosin (H&E; 200X) staining shows marked portal inflammation and ductular reaction in DDC mice, which are attenuated in the GW4064- and OCA-treated groups. Immunohistochemical staining for the macrophage marker F4/80 demonstrates increased hepatic macrophage infiltration in DDC mice, which is reduced following FXR activation. CK19 staining shows increased ductular reaction in DDC mice that is significantly diminished with FXR agonist treatment. Co-treatment with the STAT3 inhibitor C-188-9 in GW4064 treated DDC mice reversed the protective effects of GW4064. Quantification of F4/80-positive macrophages (100X) and CK19 (100X) staining is shown. *C*, unsupervised clustering of differentially expressed genes (DEGs) identified by RNAseq analysis of liver among four mouse groups: Chow, DDC, DDC/GW4064 and DDC/OCA (FDR <0.05, |log_2_FC| ≥ 1). *D*, DEGs in liver between Chow *versus* DDC mice, DDC/GW4064 *versus* DDC mice, and DDC/OCA *versus* DDC mice (FDR <0.05, |log_2_FC| ≥ 1). DDC mice show a transcriptional signature distinct from Chow mice, with significant transcriptional changes induced by treatment with both FXR agonists. *E*, gene set enrichment analysis (GSEA) identifies selected biological pathways affected in DDC-induced cholestatic injury *versus* Chow mouse liver, including oxidative phosphorylation, mitochondrial translation, NF-kB signaling, fatty acid β-oxidation, AA metabolism, macrophage migration, inflammatory signaling, TGF-beta signaling and JAK-STAT pathway activation. Bars represent normalized enrichment scores. (*F*) Gene set enrichment analysis (GSEA) of Hallmark pathways comparing DDC *versus* Chow, DDC/GW4064 *versus* DDC, and DDC/OCA *versus* DDC liver transcriptomes. Dot size represents pathway significance (−log10 FDR), and color indicates the normalized enrichment score (NES), with positive NES (*light blue*) indicating pathway enrichment and negative NES (*dark blue*) indicating pathway suppression in the indicated comparison. Data are presented as mean ± SEM (n = 3–5 mice per group). Statistical significance was determined using one-way ANOVA with appropriate multiple-comparison correction. a = *p* < 0.05 *versus* all other groups; b = *p* < 0.05 *versus* DDC mice.

To determine whether STAT3 is required for FXR-mediated hepatoprotection in this model, co-treatment with C-188-9 was performed in DDC/GW4064 mice and it partially reversed the protective effects of GW4064, as evidenced by increased inflammatory cell infiltration, F4/80-positive macrophages, and CK19-positive ductular reaction compared with the GW4064-treated group. These findings indicate that STAT3 was an apparent mediator of the anti-inflammatory and hepatoprotective effects of FXR activation during cholestatic liver injury (Fig. 1, *A* and *B*).

To identify the transcriptional programs altered in DDC mice and involved in FXR-agonist-mediated protection, we conducted bulk RNA-seq analysis of liver tissues across different experimental groups. Differential gene expression (DEG) analysis showed significant transcriptional changes during DDC-induced cholestatic injury compared to chow controls and between OCA and GW4064-treated DDC mice compared to DDC mice (Fig. 1, *C* and *D*). Volcano plot analysis further demonstrated widespread differences in gene expression among groups (Fig. S1*A*), while Venn diagram analysis identified both unique and shared genes regulated by GW4064 and OCA (Fig. S1*B*). The Venn diagram illustrates the overlap of DEGs among the DDC *versus* Chow, GW4064/DDC *versus* DDC, and OCA/DDC *versus* DDC mice. DDC feeding induced 2671 unique DEGs, while 67 genes were uniquely regulated by GW4064/DDC treatment, and 360 genes were uniquely altered by OCA/DDC treatment. There were also sets of genes shared by the four groups of mice, indicating both distinct and shared FXR-regulated transcriptional programs during cholestatic injury. Gene set enrichment analysis (GSEA) identified selected biological pathways affected in DDC-induced cholestatic injury *versus*. Chow mouse liver, including oxidative phosphorylation, mitochondrial translation, NF-kB signaling, fatty acid β-oxidation, AA metabolism, macrophage migration, inflammatory signaling, TGF-beta signaling, and JAK-STAT pathway activation (Fig. 1*E*). Treatment with the FXR agonists GW4064 and OCA broadly reversed these pathogenic pathways while restoring metabolic pathways, including fatty acid, bile acid, peroxisomal, and oxidative phosphorylation pathways. These findings indicate that FXR activation suppresses inflammatory injury while promoting metabolic reprogramming during cholestatic liver injury (Fig. 1*F*). Heatmap analysis of STAT3 target genes, *Acox1*, *Cpt1a*, *Socs1*, *Socs3*, and *Stat3*, demonstrated distinct expression patterns among Chow, DDC, DDC/GW4064, and DDC/OCA groups. Importantly, DDC feeding altered genes associated with STAT3 signaling and fatty acid oxidation, whereas treatment with FXR agonists partially reprogrammed these expression profiles, supporting a link between FXR-dependent STAT3 signaling and hepatic fatty acid metabolism (Fig. S1*C*).

### Arachidonic acid–driven oxylipin remodeling promotes liver injury and inflammatory signaling that is attenuated by FXR activation

Our transcriptomic RNA seq analyses showed enrichment of AA metabolic processes and AA acid monooxygenase in DDC *versus* Chow mice (Figs. 1*E* and 2*A*). Therefore, we hypothesized that inflammatory AA may be associated with the initiation of cholestasis and cholangiopathy. Consequently, we next performed hepatic lipidomics by LC-MS in DDC and FXR-agonist-treated mice to determine the effects of cholestasis on AA oxylpin profiles. Heatmap analysis of AA metabolites showed remodeling across experimental groups (Fig. 2*B*). DDC mice showed significant enrichment of AA and multiple AA metabolites involved in inflammatory pathways; GW4064 attenuated this enrichment in DDC mice more effectively than OCA treatment. Principal component analysis of liver metabolomics from DDC, Chow, DDC/GW4064, and DDC/OCA mice showed clear separation by PLSDA of profiles between experimental groups (Fig. S2*A*). Pathway analysis of DDC mice showed enrichment of metabolites in the AA and related fatty acid biosynthesis pathways, which was suppressed by GW4064 and, to a lesser extent, by OCA treatment (Fig. S2, *B–D*). Heatmap analysis of differentially regulated oxylipins revealed distinct clusters of AA-derived metabolites that were enriched in DDC mice and partially normalized following GW4064 (Fig. S2*E*). Together, these results demonstrate AA oxylipin remodeling in DDC mice that is partially reversed by FXR agonist treatment.

**Figure 2:**
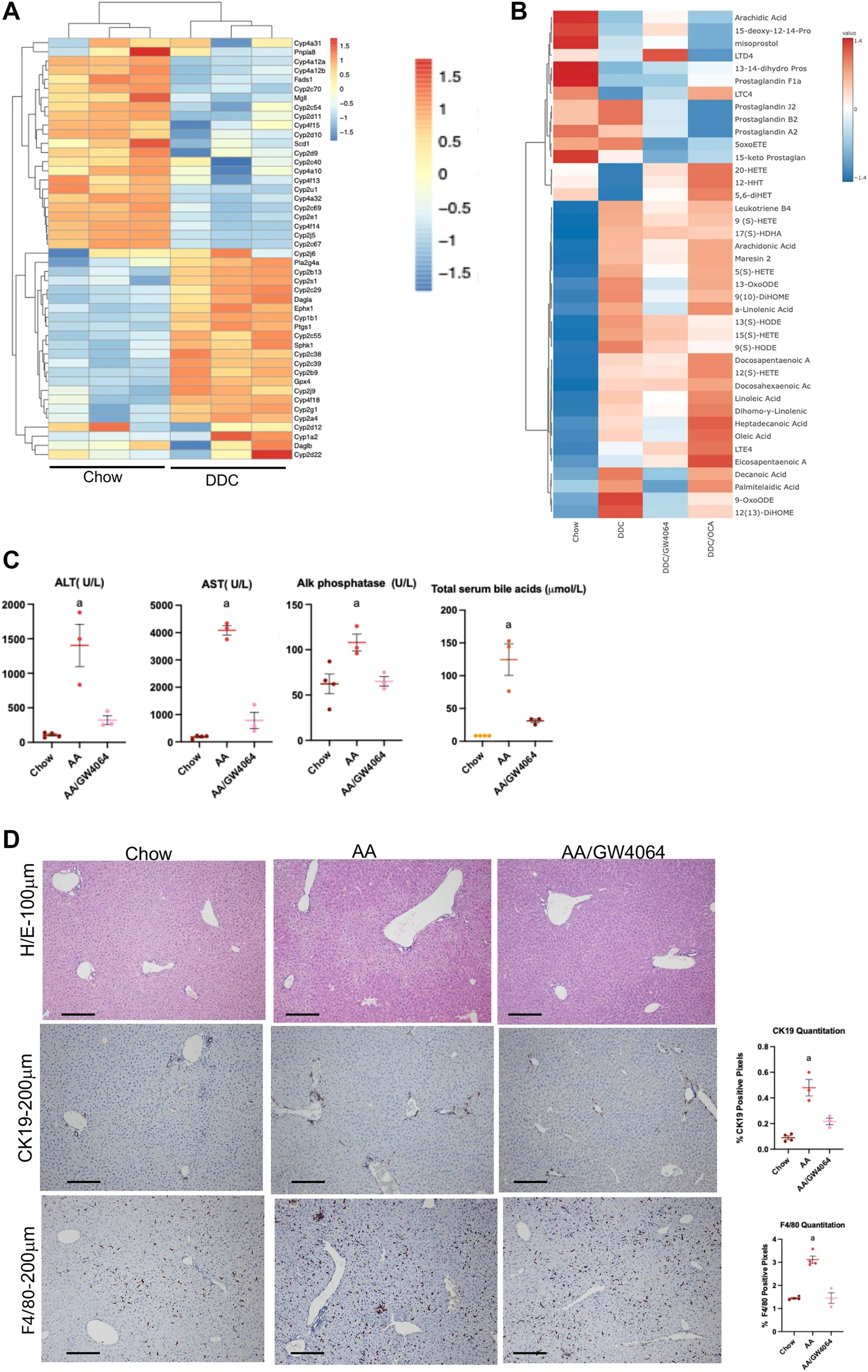

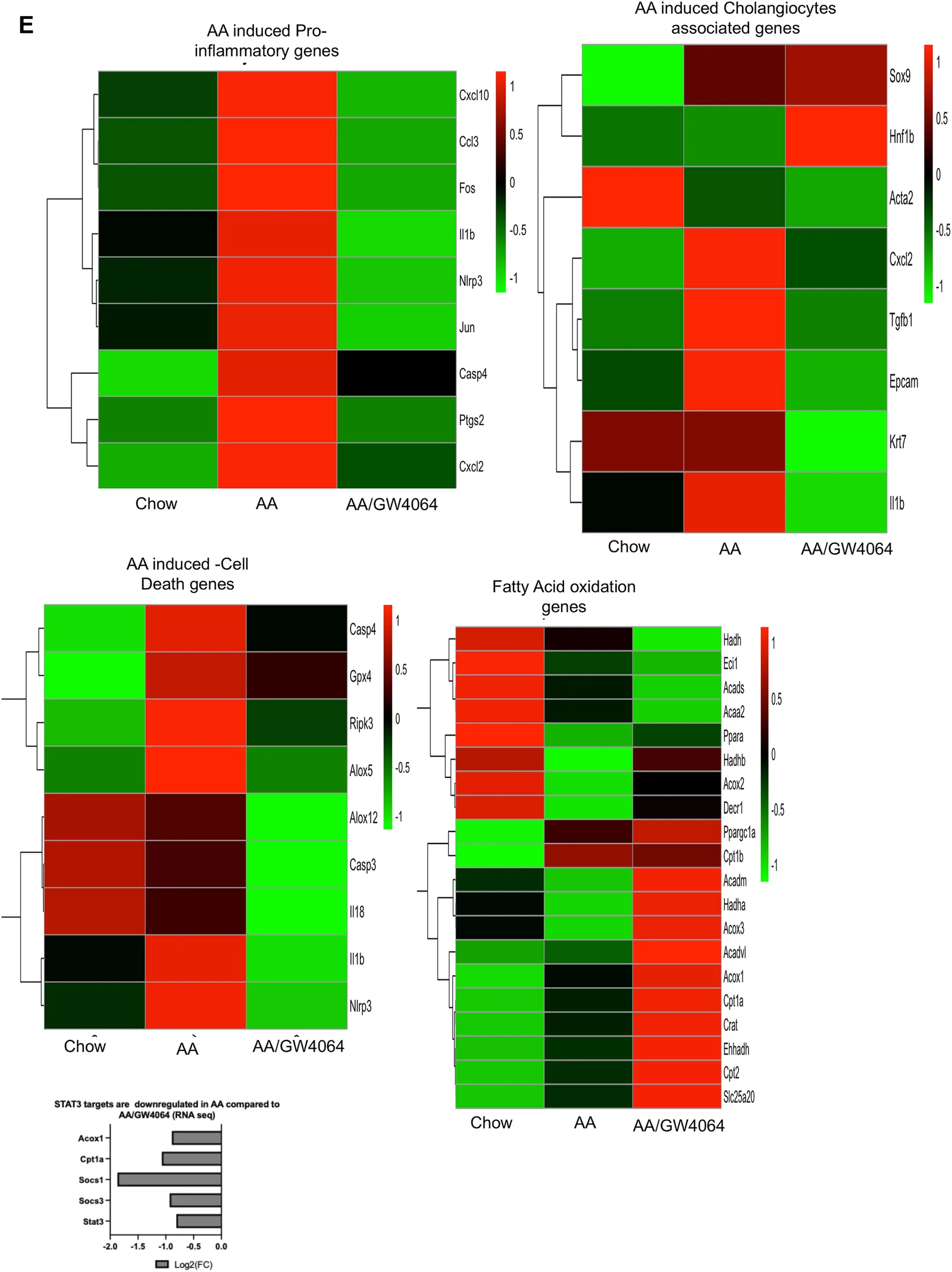

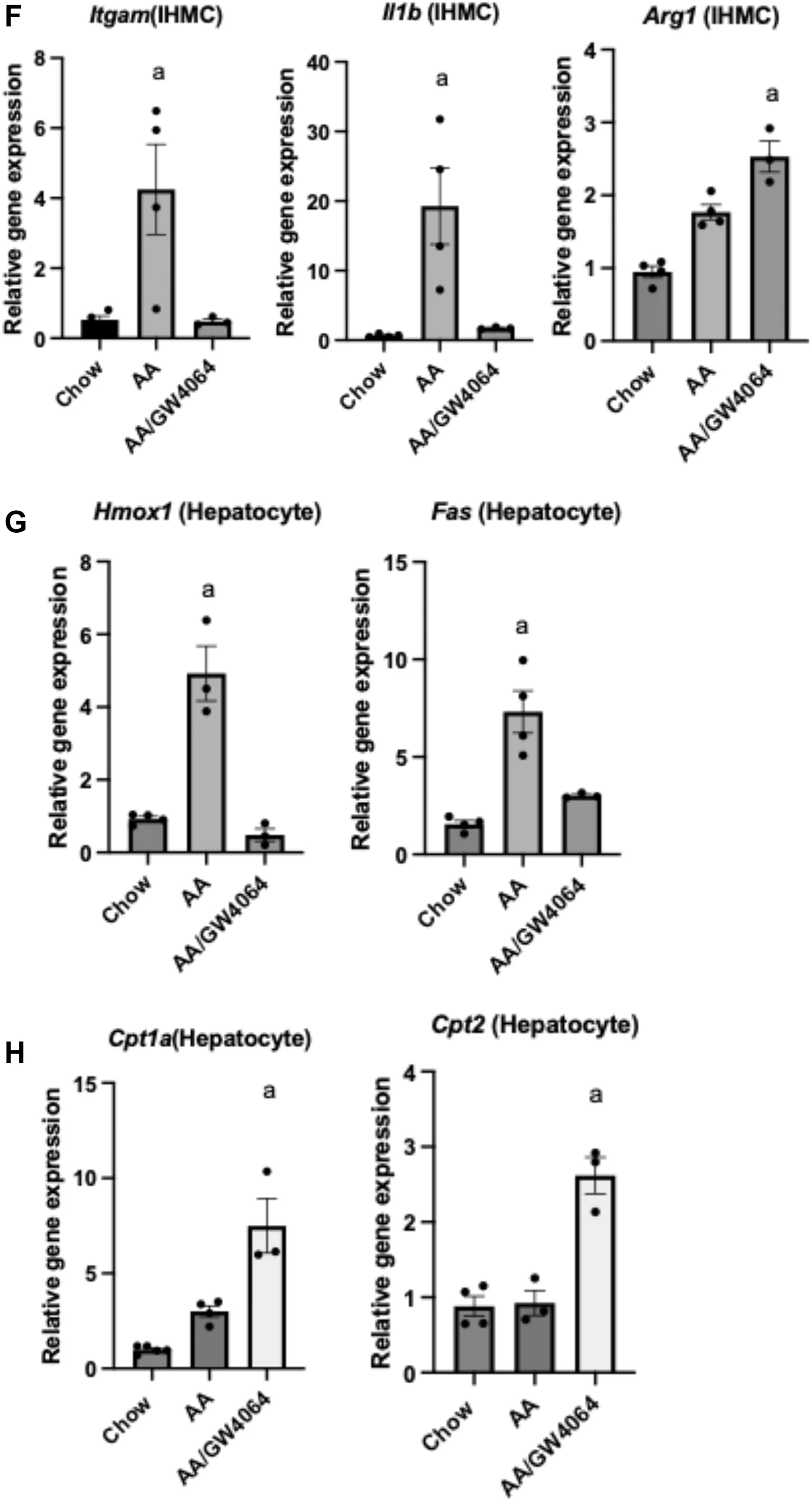

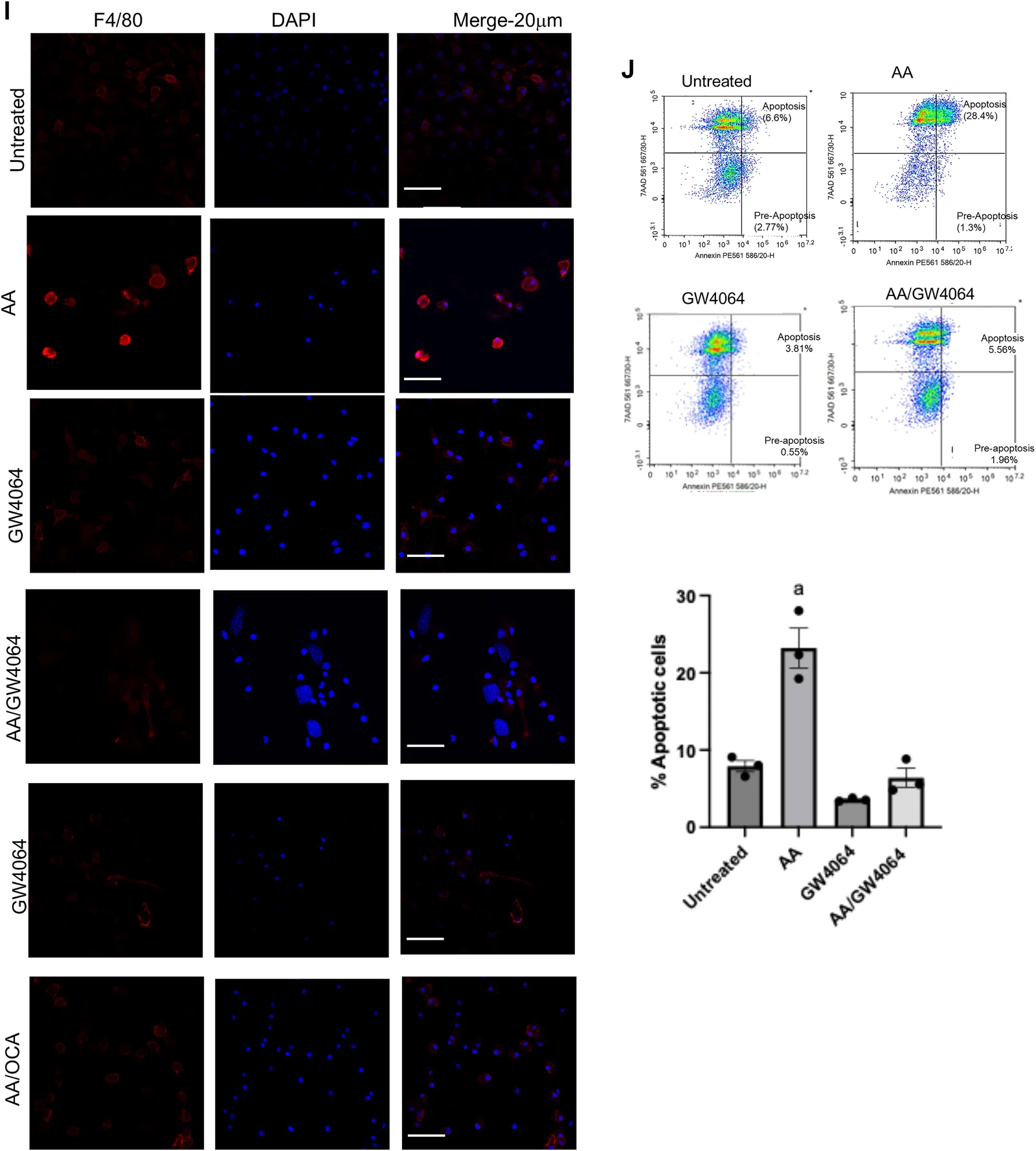
**Arachidonic acid-driven oxylipin remodeling promotes liver injury that is attenuated by FXR activation.***A*, heatmap of genes involved in arachidonic acid metabolic processes demonstrating clustering by altered hepatic expression patterns in DDC compared with Chow mice. *B*, lipidomics analysis of liver from experimental mouse groups displayed as heatmaps shows differential abundance of oxylipin metabolites across experimental groups, with increased abundance of AA-derived lipid mediators, which are remodeled with FXR agonist treatment. *C*, serum liver injury markers in Chow, AA-treated, and AA/GW4064 groups, showing evidence of significant liver injury with AA treatment and protection by GW4064. *D*, H&E (200X) staining of liver sections shows portal tract expansion and inflammation in AA-treated mice, which was reversed with GW4064 treatment. Immunohistochemical staining for CK19 and F4/80 show increased ductular responses and macrophage infiltration, respectively, following AA treatment, which were reduced by GW4064. Quantification of the IHC data for CK19 (100X) and F4/80 (100X). *E*, heatmap of RNA-seq data displaying the hepatic expression of AA-induced pro-inflammatory macrophage genes, cholangiocyte-associated genes, and cell-death-associated genes in Chow, AA and AA/GW4064mice, indicating upregulated pro-inflammatory macrophage signatures during cholestatic injury. Regulation of FAO genes in Chow, AA and AA/GW4064mice Bar plot shows Stat3-regulated AA metabolism gene are downregulated in AA compared to AA/GW4064. *F*, quantitative PCR analysis of pro-inflammatory (*Itgam*, *Il1b*) and Anti-inflammatory (*Arg1*) macrophage genes in lHMCs isolated from liver of three experimental mice groups (Chow, AA and AA/GW4064). *G*, quantitative PCR analysis of apoptotic genes in hepatocytes isolated from liver of the three experimental mice groups (Chow, AA and AA/GW4064). *H*, quantitative PCR analysis of FAO genes in hepatocytes isolated from liver of the three experimental mice groups (Chow, AA and AA/GW4064). *I*, immunofluorescence pro-inflammatory pan macrophage marker (F4/80) analysis of Chow mice cultured bone marrow-derived macrophages (BMDMs) treated with AA overnight, with or without FXR agonists (GW4064 or OCA). *J*, flow cytometric analysis of Annexin V as a measure of apoptosis in cultured Huh7 cells that were treated overnight with arachidonic acid (AA) in the presence or absence of FXR agonist, GW4064, demonstrating increased apoptotic cells following AA exposure which reduced to control levels by GW4064. Data are presented as mean ± SEM (n = 3–5 mice per group; n = 3 independent biological replicates for cell culture experiments). Statistical significance was determined using one-way ANOVA with Tukey’s correction for multiple comparisons and two tailed unpaired Student’s *t* test. a = *p* < 0.05 *versus* all other groups.

Because AA metabolism pathways were markedly enriched in DDC mice, subsequent experiments focused on the role of AA in the induction of liver injury (15, 16). Since GW4064 treatment in DDC mice had a more robust effect than did OCA on reversing the metabolite pattern observed in DDC mice, we focused further analysis on the role of GW4064 in this pathway. Chow mice sacrificed 4 h after i.p. AA injection had significant elevations of serum liver injury markers (ALT, AST, total bile acids, and alkaline phosphatase) compared to Chow controls (Fig. 2*C*) as well as ductular reaction (Fig. 2*D*) and macrophage infiltration (Fig. 2*D*). These liver chemistries and histological alterations were reversed in GW4064-treated AA mice (Fig. 2, *C* and *D*). Bulk RNA-seq analysis showed that AA mice exhibited upregulation of pro-inflammatory genes, cholangiocyte markers, and cell death–related pathways, along with downregulation of fatty acid–related genes, compared with both Chow and AA/GW4064 mice. These findings suggest that AA induces a transcriptional program resembling cholestatic DDC injury, whereas GW4064 partially reverses this signature (Fig. 2*E*). Consistent with these transcriptomic findings, qPCR analysis on IHMCs isolated from AA mice confirmed significant upregulation of macrophage recruitment and activation markers (*Itgam*/CD11b) and the inflammatory cytokine *Il1b* in AA mice (Fig. 2*F*). Treatment with GW4064 significantly reduced the expression of these pro-inflammatory markers and upregulated the anti-inflammatory marker *Arg1* in IHMC and the FAO genes *Cpt1a and Cpt2* in hepatocytes (Fig. 2, *F* and *H*). Similarly, FXR agonist treatment with GW4064 decreased the expression of cell death markers in hepatocytes isolated from AA mice (Fig. 2*G*), indicating that FXR signaling promotes a molecular shift toward anti-inflammatory pathways.

To determine whether lipid mediator signaling could similarly activate macrophages, cultured mouse bone marrow–derived macrophages (BMDMs) were treated with AA overnight. AA exposure increased F4/80 macrophage staining measured by confocal microscopy, whereas treatment with FXR agonists GW4064 or OCA attenuated these responses (Fig. 2*I*). Because treatment of mice with AA activated cell death pathways, we next treated cultured Huh7 cells with AA overnight in the presence or absence of GW4064, performed FACS analysis using Annexin V staining, and found that AA markedly increased apoptotic cell populations compared to untreated control cells (Fig. 2*J*) and that GW4064 significantly reduced AA-induced apoptosis, indicating that FXR activation directly protects hepatocytes from AA-mediated cell death.

Bulk RNA-seq analysis revealed that STAT3 target genes in the liver were significantly downregulated in the AA group compared to AA/GW4064 group, showing that FXR activation upregulated STAT3-dependent signaling *in vivo* in the liver (Fig. 2*E*). We next examined the effect of GW4064 on STAT3 targets expression in liver. (Fig. 2*E*). To further understand how FXR signaling influences hepatic AA metabolism during cholangiopathy, we analyzed RNA-seq data and examined AA-related genes enriched in the DDC/GW4064 group. Heatmap analysis showed strong activation of AA-COX pathway genes in DDC mouse liver which was further enhanced after FXR activation (Fig. S2*E*). Notably, expression of several STAT3-associated cytochrome P450 enzymes, including *Cyp2c29*, *Cyp2b10*, *Cyp1a2*, *Cyp3a11*, *Cyp2c38*, *Cyp2c55*, *Cyp4a14*, *and Cyp2j6*, was significantly upregulated in the GW4064-treated DDC group compared to both Chow and DDC mice (Fig. S2*E*). We hypothesized that AA metabolism was associated with anti-inflammation in this model. As a representative AA downstream prostaglandin’s effect on macrophage expression, we incubated Raw 264.7 cells with prostaglandin D2 overnight and found that expression of anti-inflammatory genes, including *Klf4*, *Retnlg*, *Clec10a1*, and *Maf*, was significantly increased by prostaglandin D2 treatment. These data support the notion that FXR activation can ultimately lead to transcriptional programs that link anti-inflammatory signaling to AA metabolic reprogramming (Fig. S2*F*).

### FXR activation suppresses hepatocyte apoptosis and induces STAT3 signaling during cholestatic liver injury

Given that cholestatic liver injury is associated with hepatocyte apoptosis and inflammatory signaling, we next examined whether FXR activation influences protection against hepatocyte death during DDC-induced injury (8). Flow cytometric analysis of isolated hepatocytes showed a significant increase in Annexin V-positive apoptotic cells in DDC mice compared to Chow controls (Fig. 3*A*). Heatmap analysis demonstrated significant upregulation of expression of apoptosis-related genes in the livers of DDC mice (Fig. S3*A*). Treatment with GW4064 or OCA significantly reduced DDC-induced hepatocyte apoptosis and apoptosis-related gene expression in the livers of DDC mice, as analyzed by heatmap clustering (Figs. 3*A* and S3*A*). Consistent with these results, flow cytometry of hepatocyte populations revealed a significant increase in CD95/FAS-positive hepatocytes in DDC mice, which were reduced by both GW4064 and OCA treatment (Fig. 3*B*). Similarly, measurement of caspase-8 and caspase-3/7 activity confirmed increased apoptotic signaling in hepatocytes from DDC livers, which was significantly reduced after FXR agonist treatment (Fig. 3, *C* and *D*).

**Figure 3:**
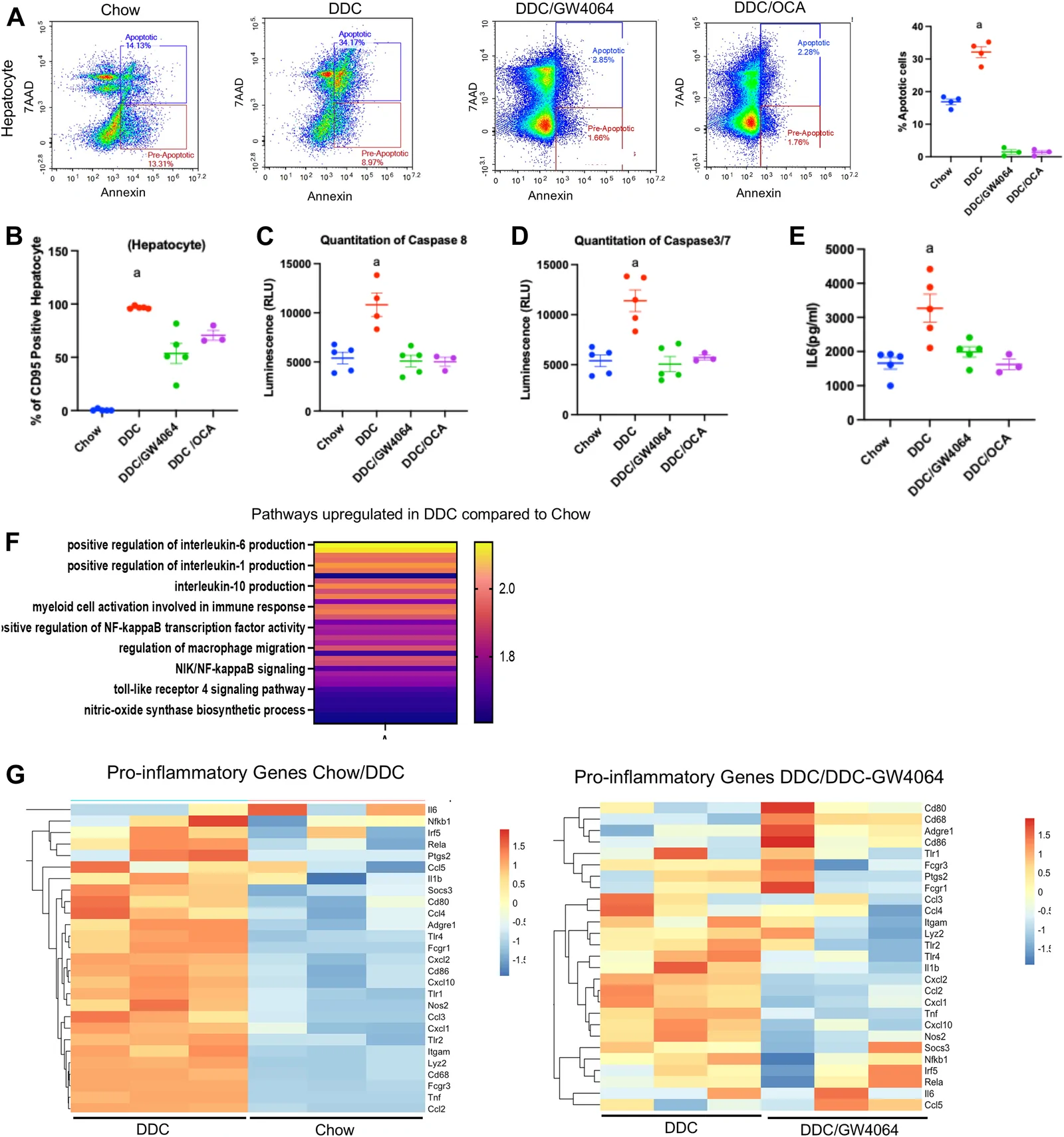

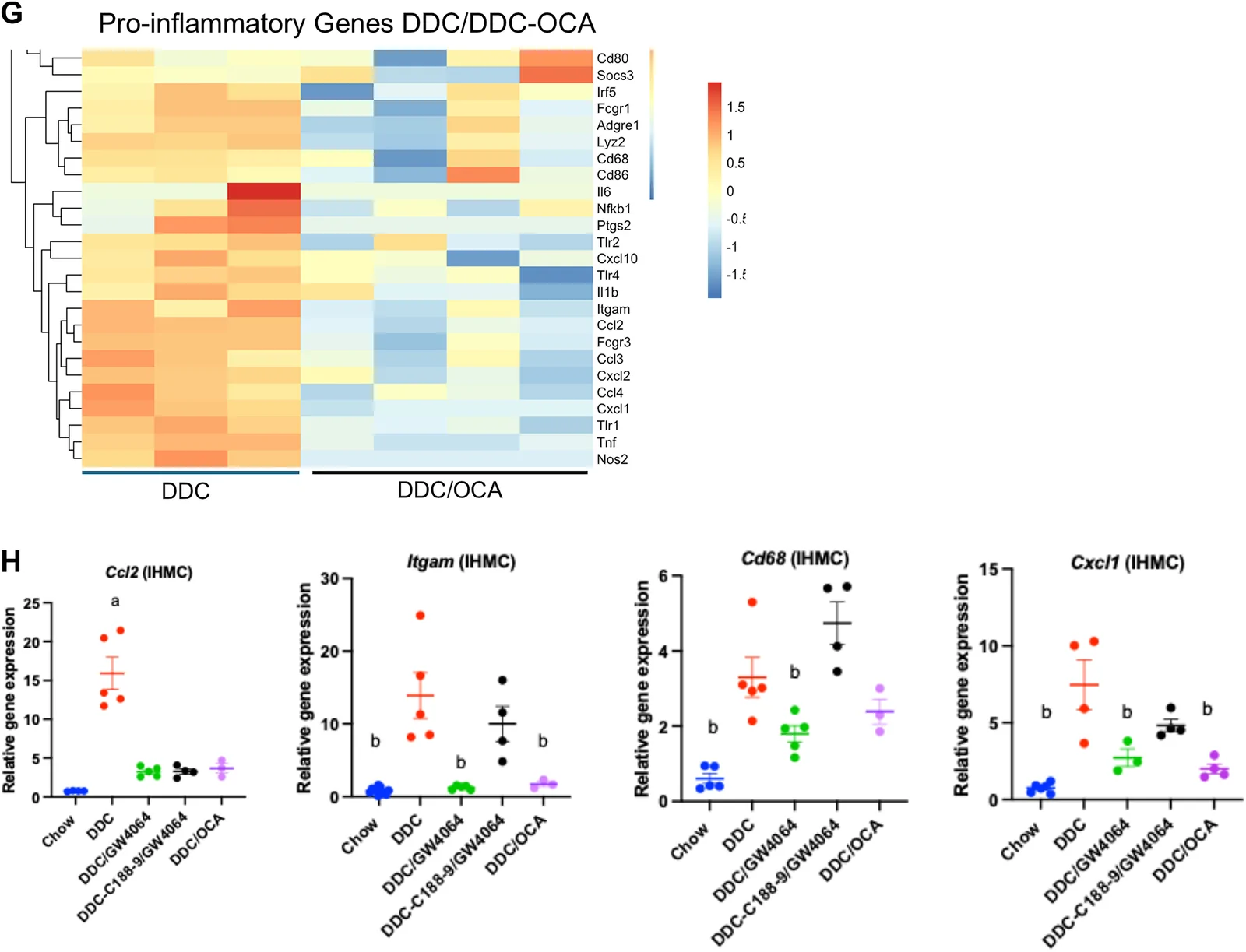
**FXR activation reduces hepatocyte apoptosis during cholestatic injury.***A*, flow cytometric analysis for apoptosis using Annexin V staining of hepatocytes isolated from Chow, DDC, DDC/GW4064, and DDC/OCA mice. DDC feeding significantly increased the percentage of apoptotic hepatocytes, which was reduced following FXR agonist treatment. *B*, quantification of flow cytometric analysis of CD95/FasR in isolated hepatocytes. DDC treatment markedly increased CD95-positive hepatocytes compared with Chow controls, whereas GW4064 and OCA significantly reduced CD95 expression. Quantification for groups of n = 3 to 5 mice per group. *C* and *D*, Caspase-8 and Caspase-3/7 activity in hepatocytes isolated from the four groups of mice, demonstrating increased apoptotic signaling in DDC-treated mice that is attenuated by FXR agonists. *E*, serum IL-6 levels across experimental groups, showing increased IL-6 levels in serum from DDC mice that was reduced following FXR agonist treatment in DDC mice. *F*, pathway enrichment analysis of RNA-seq data from mouse liver presented as ranking of the inflammatory pathways upregulated in DDC mice compared to Chow mice. *G*, heatmap of RNA-seq data displaying the hepatic expression of selected pro-inflammatory macrophage genes in Chow and DDC mice, indicating clustering of upregulated pro-inflammatory macrophage signatures during cholestatic injury. Heatmap of RNA-seq data displaying the hepatic expression of selected pro-inflammatory macrophage genes in DDC/GW4064 *versus* DDC mice. Heatmap of RNA-seq data displaying the hepatic expression of selected pro-inflammatory macrophage genes in DDC/OCA *versus* DDC mice. *H*, quantitative PCR analysis of pro-inflammatory macrophage genes in lHMCs isolated from liver of the five experimental groups, including *Ccl2*, *Itgam*/*CD11b*, *Cd68*, and *Cxcl1*. Data in (*A*) to (*E*) and (*H*) are presented as mean ± SEM (n = 3–5 mice per group). Statistical significance was determined using one-way ANOVA with Tukey’s correction for multiple comparisons, a = *p* < 0.05 *versus* all other groups.

STAT3 signaling pathway was significantly enriched in DDC mouse livers (Fig. 1*E*), as indicated by activation of inflammatory cytokine IL6 signaling during cholestatic injury. Supporting this concept, serum IL-6 levels were notably higher in DDC mice but decreased following GW4064 and OCA administration (Fig. 3*E*). Further pathway enrichment analysis of liver from DDC compared to Chow mice demonstrated the activation of inflammatory pathways in DDC mice, including IL-6 production, NF-κB signaling, macrophage migration, and Toll-like receptor signaling (Fig. 3*F*).

Because hepatic macrophages are key players in cholestatic inflammation, we next examined whether FXR signaling influences macrophage polarization during DDC-induced liver injury. Transcriptomic analysis showed strong activation of pro-inflammatory macrophage programs in DDC-treated livers. Clustering analysis of gene expression heatmaps of macrophage-related pro-inflammatory genes demonstrated significant upregulation of inflammatory macrophage signatures in DDC mice compared to Chow controls (Fig. 3*G*). Treatment with the FXR agonists GW4064 or OCA in DDC mice significantly reduced the expression of pro-inflammatory genes (Fig. 3*G*). Consistent with these transcriptomic findings, qPCR analysis of mouse IHMCs confirmed significant upregulation of macrophage recruitment and activation genes (*Ccl2*, *Itgam*/CD11b, *Cd68*, *Cxcl1*, and *Cd80*) and in DDC mouse liver, of the inflammatory cytokine *Il1b* (Figs. 3*H* and S3*G*), all of which were suppressed by FXR agonist treatment (Fig. 3*H*). In addition, co-treatment with the STAT3 inhibitor, C-188-9, with GW4064 further upregulated the transcriptomic response of *Itgam*, *CD68* and *Cxcl1*, indicating that STAT3 contributes to the FXR-mediated anti-inflammatory gene regulation during cholestatic liver injury (Fig. 3*H*).

Heat map analysis of anti-inflammatory gene expression revealed no significant differences in pairwise comparisons among the groups (Fig. S3, *D* and *F*). Nevertheless, treatment with the FXR agonist was associated with increased expression of anti-inflammatory/alternative macrophage markers, including *Cd163*, *Retnlg*, and *Chil3* (Fig. S3*G*). Together, these findings are consistent with FXR signaling promoting a molecular shift toward anti-inflammatory macrophage polarization.

### FXR activation suppresses TGF-β–driven fibrotic remodeling and bile acid dysregulation during cholestatic injury

Our results demonstrated that FXR signaling regulates macrophage polarization and metabolic reprogramming in DDC mice and enrichment of TGF-β signaling in DDC mice. Since cholestasis is closely associated with progressive fibrosis and activation of TGF-β signaling, we next examined the TGF pathway as a potential regulatory axis linking cholestatic injury to fibrogenic responses (17). Transcriptomic analysis revealed upregulation of TGF-β–responsive genes (extracellular matrix remodeling, inflammatory signaling, and fibrogenesis pathways) in livers from DDC mice (Fig. 4*A*), which were suppressed by treatment with the FXR agonists GW4064 or OCA (Fig. 4, *B* and *C*). Histological analysis further supported these transcriptomic results. Liver sections from DDC mice displayed periportal fibrosis, which was prevented by FXR agonist treatment (Fig. 4*D*). Consistent with these findings, qPCR analysis demonstrated significant upregulation of fibrogenesis-associated genes (*Adam1*, *Spp1*, *S100a4*, *Timp2*, *Mmp13*, *and Col1a1*) in livers from DDC mice compared with Chow controls (Fig. 4*E*), which was prevented by both GW4064 and OCA treatments. Co-treatment with the STAT3 inhibitor C-188-9 and with GW4064 partially attenuated these protective effects of GW4064 in mouse liver (Fig. 4*E*), indicating that STAT3 contributed to the antifibrotic actions of FXR activation in this model.

**Figure 4:**
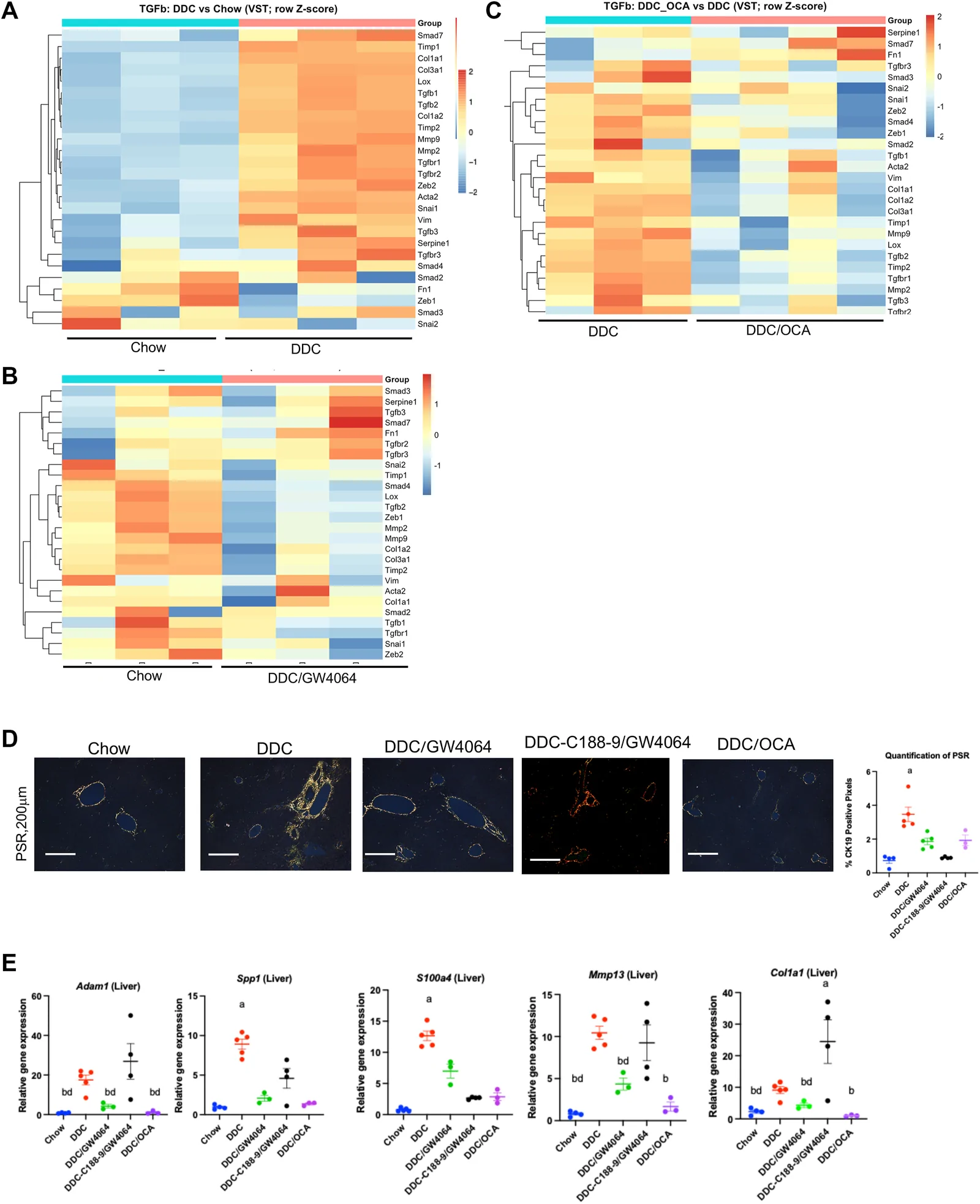
**FXR activation suppresses expression of TGF-β associated genes and fibrotic remodeling in DDC mouse liver.***A*, RNA-seq heatmap illustrating the expression of TGF-β–associated genes in Chow and DDC mouse livers. *B* and *C*, heatmaps displaying effects of FXR agonists on transcriptional changes in TGF-β-associated genes. *D*, representative images of PSR stained liver using polarized light microscopy demonstrating portal fibrotic remodeling in DDC mice which was prevented in FXR agonist treated mice. *E*, qPCR analysis of fibrosis-related genes (*Adam1*, *Spp1*, *S100a4*, *Mmp13*, *Col1a1*) from liver of experimental groups, showing upregulation in DDC mice which was suppressed in DDC mice treated with FXR agonists. Co-treatment with the STAT3 inhibitor C-188-9 attenuated these protective effects in DDC mice treated with GW4064. Data are presented as mean ± SEM (n = 3–5 mice per group). Statistical significance was determined using one-way ANOVA with appropriate multiple-comparison correction. a = *p* < 0.05 *versus* all other groups; b = *p* < 0.05 *versus* DDC mice.

Metabolomic analysis of liver bile acid composition revealed that cholestatic injury in DDC mice was associated with significant changes in hepatic bile acid composition. PCA analysis demonstrated a clear separation of bile acid profiles among Chow, DDC, and GW4064-treated DDC mice (Fig. S4*A*) generated from metabolomic analysis. Heatmap analysis showed elevated primary bile acids in DDC compared to Chow mice, which was attenuated by GW4064 treatment (Fig. S4*B*); fold-change analysis between groups further supports these differences (Fig. S4*C*).

Together, these findings demonstrate that upregulation of FXR signaling suppresses TGF-β–driven fibrotic gene programs and restores bile acid homeostasis during cholestatic injury. When integrated with earlier results, these data support a model in which FXR activation suppresses AA-driven pro-inflammatory signaling and ultimately limits progression of cholangiopathy and fibrotic remodeling.

### FXR activation induces STAT3 signaling which attenuates cholestatic liver injury

Previously, we reported that FXR agonist GW4064 induced the STAT3 pathway, which was protective in another mouse model of cholestasis, parenteral nutrition-associated cholestasis (PNAC) (8). We now show that the JAK/STAT signaling pathway was significantly enriched in DDC mouse livers (Fig. 1*E*). Heat map analysis also showed that expression of genes involved in the IL-6-STAT3 signaling pathway was significantly enriched in DDC mouse livers (Fig. 5*A*). ChIP analysis of mouse liver showed mild enrichment of FXR binding at the *Stat3* promoter region in DDC mice, which was further increased by FXR agonist treatment and was paralleled by mRNA expression of *Stat3* (Fig. 5*B*). Conversely, Western blot analysis revealed decreased phosphorylation of STAT3 in DDC mouse livers, while treatment with GW4064 or OCA increased pSTAT3 (at position Lys 705) compared to DDC mouse livers (Fig. 5*C*). Since STAT3 activation is protective in other cholestatic mouse models (8), these data are consistent with STAT3 phosphorylation playing a protective role in DDC mice.

**Figure 5:**
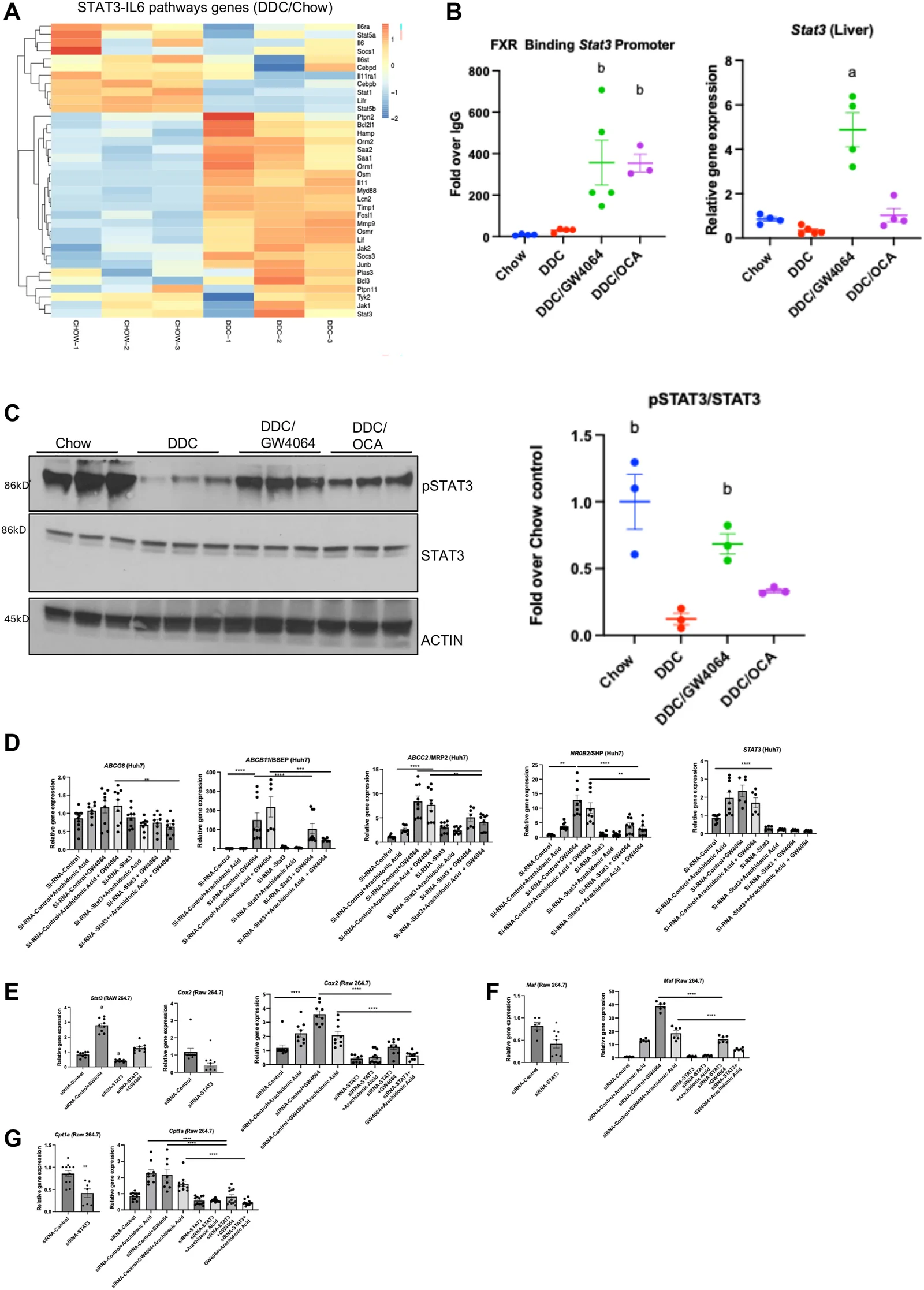

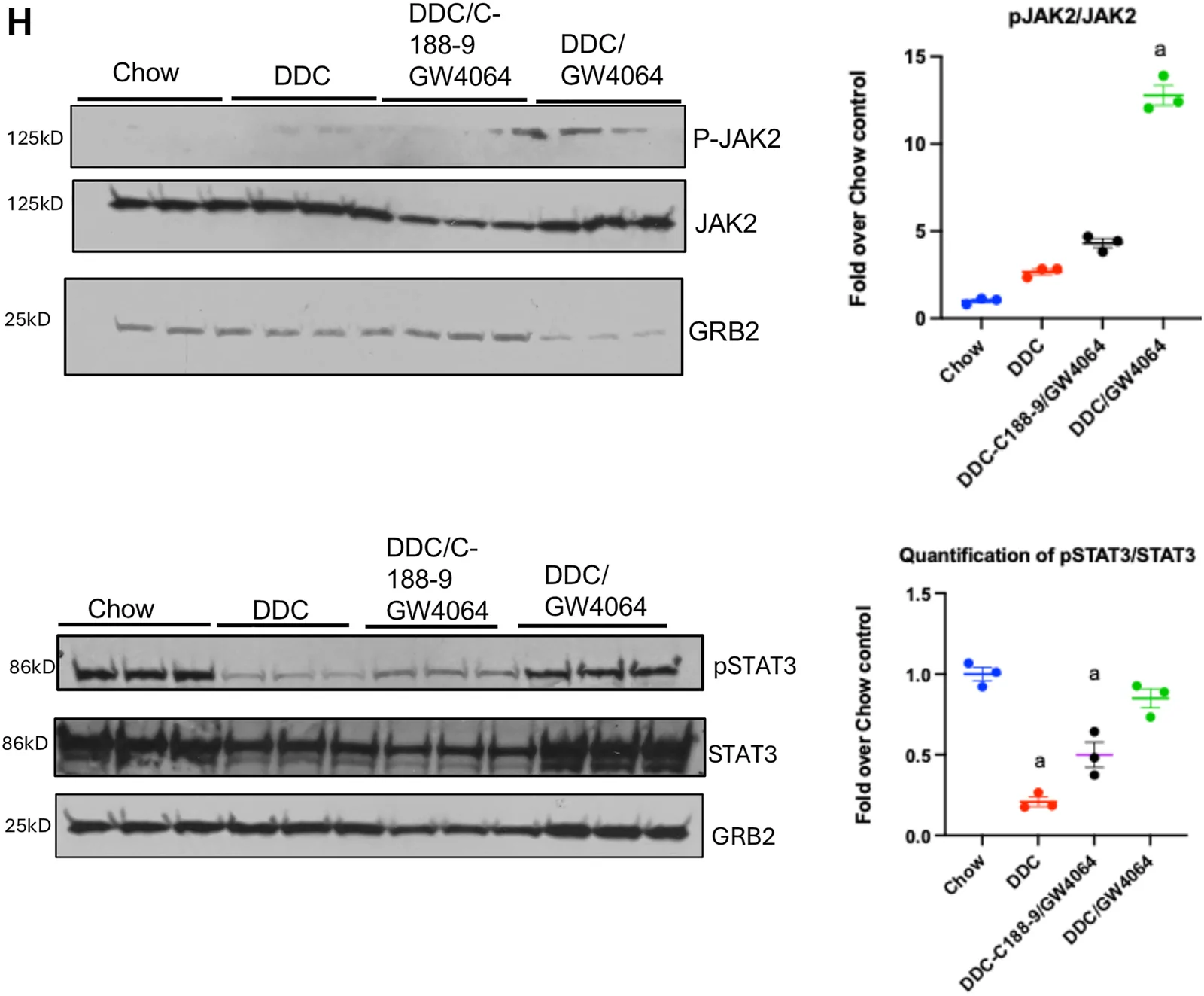
**FXR agonist induced STAT3 pathway.***A*, heatmap of RNA-seq expression of genes associated with JAK-STAT3 in DDC mouse liver and Chow. *B*, chromatin immunoprecipitation analysis showing that FXR binding at the *Stat3* promoter region was increased with FXR treatment in chronic DDC mice. *C*, Western blot analysis of phosphorylated STAT3 (pSTAT3) and total STAT3 in liver tissues from experimental mice. Quantification of the pSTAT3/STAT3 ratio is shown on the *right*. *D*, gene expression analysis in Huh7 overnight cultured cells exposed to arachidonic acid. Showing mRNA expression of bile acid transporter and nuclear receptor target genes (*STAT3*, *ABCB11*/BSEP, *ABCC2*/MRP2, *NR0B2*/*SHP*, *ABCG8*) following FXR activation by GW4064 or STAT3 knockdown by siRNA. *E*, gene expression analysis of *STAT3* in Raw 264.7 macrophages cultured overnight treated with AA and FXR agonist (GW4064) with or without STAT3 knockdown by siRNA. Expression of *Cox2 i*n Raw 264.7 macrophages under the indicated conditions, demonstrating that STAT3 knockdown reduced Cox2 expression, that GW4064 induced *Cox2* expression but that siRNA STAT3 knockdown prevented the induction of *Cox2* by GW4064. These results were the same in the presence or absence of AA. Expression of *Cox2 i*n Raw 264.7 macrophages under the indicated conditions, demonstrating that STAT3 knockdown reduced Cox2 expression, that GW4064 induced *Cox2* expression but that siRNA STAT3 knockdown prevented the induction of *Cox2* by GW4064. These results were the same in the presence or absence of AA. *F*, expression of macrophage activation marker *Maf* in cultured Raw 264.7 macrophages overnight under conditions of arachidonic acid stimulation, FXR agonist (GW4064) exposure and STAT3 knockdown by siRNA. Raw 264.7expression of *Maf* was dependent on STAT3 signaling under all conditions. *G*, functional validation experiments of STAT3 regulation of *Cpt1a* in Raw 264.7 macrophages exposed to AA demonstrate that 1) siRNASTAT3 downregulates *Cpt1*a expression, 2) increased *Cpt1a* expression is present upon AA or GW4064 exposure, and 3) that knockdown of *Stat3* suppresses expression of *Cpt1a* in cells exposed to either AA or GW4064 or both. *H*, Western blot analysis of phosphorylated JAK2 (pJAK2) and total JAK2 and phosphorylated STAT3 (pSTAT3) and total STAT3 in liver tissues from experimental mice. Quantification of the pJAK2/JAK2 and pSTAT3/STAT3 ratio is shown on the right. Data are presented as mean ± SEM (n = 3–5 mice per group, and three independent cell culture experiments including technical replicates). Statistical significance was determined using one-way ANOVA with appropriate multiple-comparison correction. a = *p* < 0.05 *versus* all other groups; b = *p* < 0.05 *versus* DDC mice; d = *p* < 0.05 *versus* DDC-C-188/GW4064 mice. For *D–G ∗p* < 0.05 *by 1*-*way ANOVA*; *∗p* < 0.05, *∗∗p* < 0.01, *∗∗∗∗p* < 0.0001.

In Figure 2, we showed that GW4064 treatment attenuated AA-induced hepatocyte injury. To evaluate whether FXR activation by GW4064 was dependent on STAT3 signaling in the presence of AA, we conducted gene expression analysis in cultured Huh7 cells treated with AA and GW4064 with or without STAT3 siRNA knockdown. (Fig. 5*D*). GW4064 induced FXR target gene expression (*ABCB11*, *ABCC2*, *NROB2*, *ABCG8*) in the presence of AA, and knockdown of STAT3 dampened the GW4064 induction effect on these genes. These findings support STAT3 playing a major role in modulating the FXR-dependent transcriptional regulation under AA-induced inflammatory conditions.

Because AA metabolism produces prostaglandins *via* cyclooxygenase (COX) pathways, we next examined expression of the STAT3 target *Cox2* in Raw 264.7macrophages. GW4064 exposure significantly increased *Cox2* expression, while *Stat3* knockdown markedly reduced this induction (Fig. 5*E*). When STAT3 was inhibited by siRNA, AA and GW4064 failed to induce *Cox2* expression. Thus, STAT3 signaling mediates the upregulation of *Cox2* expression by both FXR agonist and AA treatments. Similarly, the anti-inflammatory gene *Maf* and the FAO gene *Cpt1a* exhibited expression patterns comparable to *Cox2* upon STAT3 inhibition by siRNA, both in the presence and absence of AA and GW4064, administered alone or in combination (Fig. 5, *F* and *G*).

Co-administration of C-188-9 did not completely abolish the biochemical protection conferred by GW4064, suggesting that FXR activation also engages STAT3-independent protective mechanisms. However, STAT3 inhibition partially reversed key molecular effects of GW4064. C-188-9 targets the STAT3-SH2 domain and disrupts STAT3 Tyr705 phosphorylation, homodimerization, and subsequent transcriptional activation. Its partial attenuation of GW4064-mediated protection therefore supports a functional role for STAT3 activation in FXR-mediated hepatoprotection. Although total STAT3 protein abundance remained unchanged, GW4064 increased JAK2 phosphorylation together with STAT3 Tyr705 phosphorylation, suggesting activation of the upstream JAK2-STAT3 pathway. However, C-188-9 attenuated the protective effects of GW4064, supporting a functional requirement for STAT3 activation in FXR-mediated hepatoprotection (Fig. 5*H*). Together, these findings indicate that STAT3 signaling contributes to the FXR-mediated immunometabolism response during DDC-induced cholestatic injury, particularly through regulation of inflammatory, fibrotic, and fatty acid oxidation pathways.

Given our earlier findings demonstrating that FXR regulates STAT3 signaling, we next assessed whether STAT3 activity influences macrophage-driven inflammatory responses *in vivo*. Pharmacological inhibition of STAT3 using the selective inhibitor C-188-9, administered *via* daily i.p. injections for 3 days in Chow mice, resulting in a marked increase in hepatic macrophage infiltration and ductular reaction. This was evidenced by enhanced F4/80 and CK19 staining in C-188-9–treated mice compared to controls (Fig. S5*B*), although liver blood tests were not altered (Fig. S5*A*). Furthermore, gene expression analysis in IHMCs revealed increased mRNA levels of pro-inflammatory macrophage markers, including *Cxcl1*, *Adgre1*/*F4*/*80*, *Ccl2*, and *Itgam/CD11b* (Fig. S5*C*), To determine if STAT3 inhibition affects the interaction between macrophages and cholangiocytes, we co-cultured THP-1 cells (bottom well) with H69 cells (upper well) that were exposed to AA and with or without C188-9. STAT3 inhibition upregulated expression of *KRT19*, *CCL2*, *and IL1B* in H69 cells in the presence of AA (Fig. S5*D*) and similarly upregulated pro-inflammatory cytokine genes *CCL2*, *IL1B*, *and CXCL1* in the THP-1 cells under the same conditions. Thus, inhibiting STAT3 signaling activated macrophage pro-inflammatory polarization and infiltration. Together, these findings suggest that FXR signaling through STAT3 reduces cholestatic and AA-driven inflammatory macrophage activation and promotes anti-inflammatory polarization.

### Pharmacologic STAT3 activation attenuates cholestatic injury, inflammatory and fibrotic signaling, and upregulates fatty acid oxidation genes in DDC mice

We next investigated whether pharmacological activation of STAT3 could directly reduce cholestatic injury. DDC mice were treated with the STAT3 activator colivelin intravenously while on the DDC diet for 14 days. Liver cholestatic markers (alkaline phosphatase, total bilirubin, and total serum bile acid), which were markedly raised in DDC mice (Fig. 1*A*), were significantly reduced by colivelin treatment, while AST and ALT trended to be lower (Fig. 6*A*). Histological analysis further supported these findings. The significant macrophage infiltration and ductular reaction in DDC mice were markedly reduced by colvelin treatment (Fig. 6*B*). At the molecular level, colivelin restored hepatocyte mRNA expression of hepatocellular bile transporters (*Abcb11* (BSEP), *Nr0b2* (SHP), and *Abcc2* (MRP2)) compared to DDC mice (Fig. 6*C*). In line with enhanced metabolic reprogramming, STAT3 activation significantly upregulated genes in hepatocytes involved in FAO (*Aco2*, *Acox1*, *Cpt1a*, *Cpt1b*, and *Hadhb*) (Fig. 6*D*).

**Figure 6:**
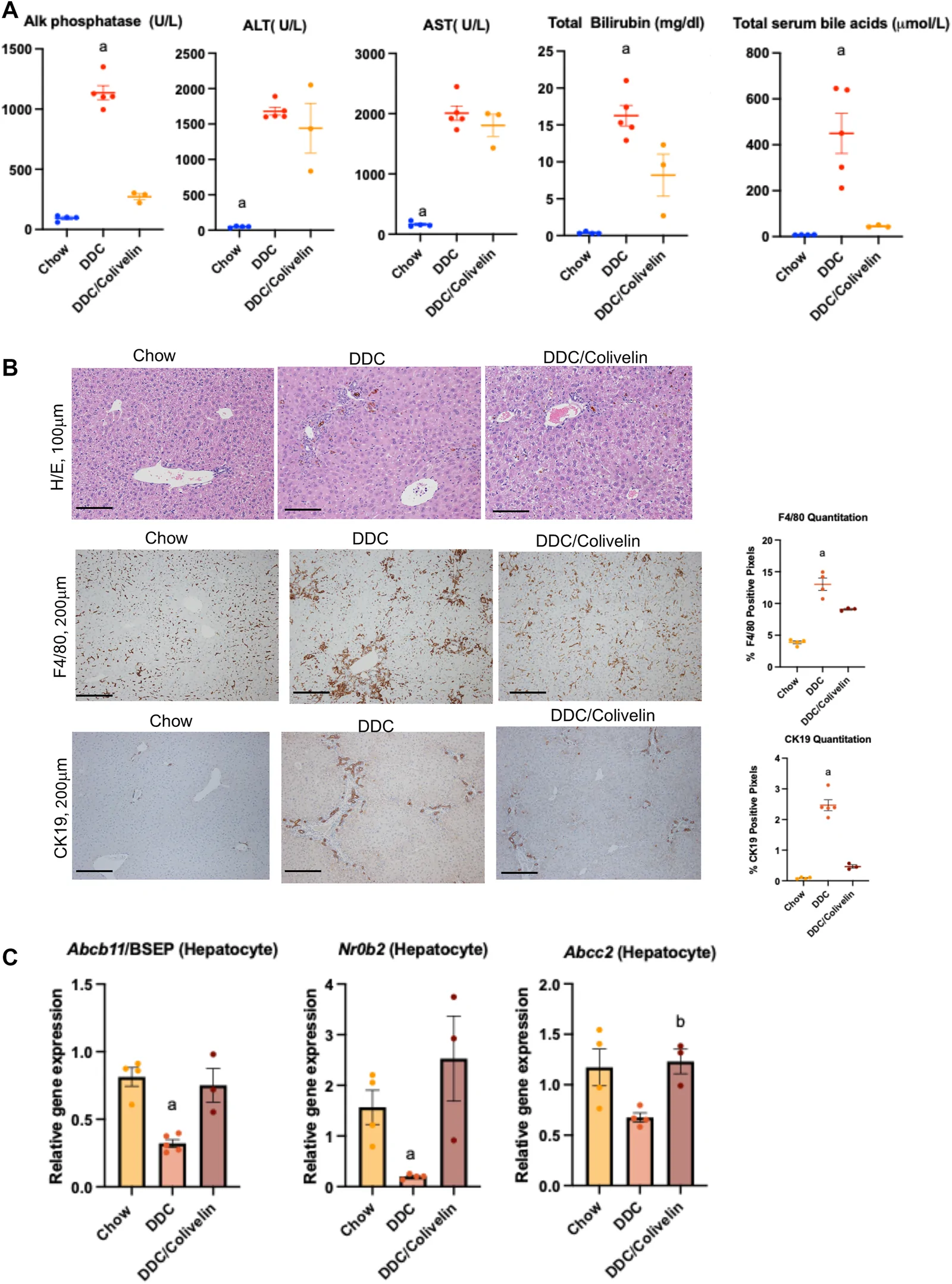

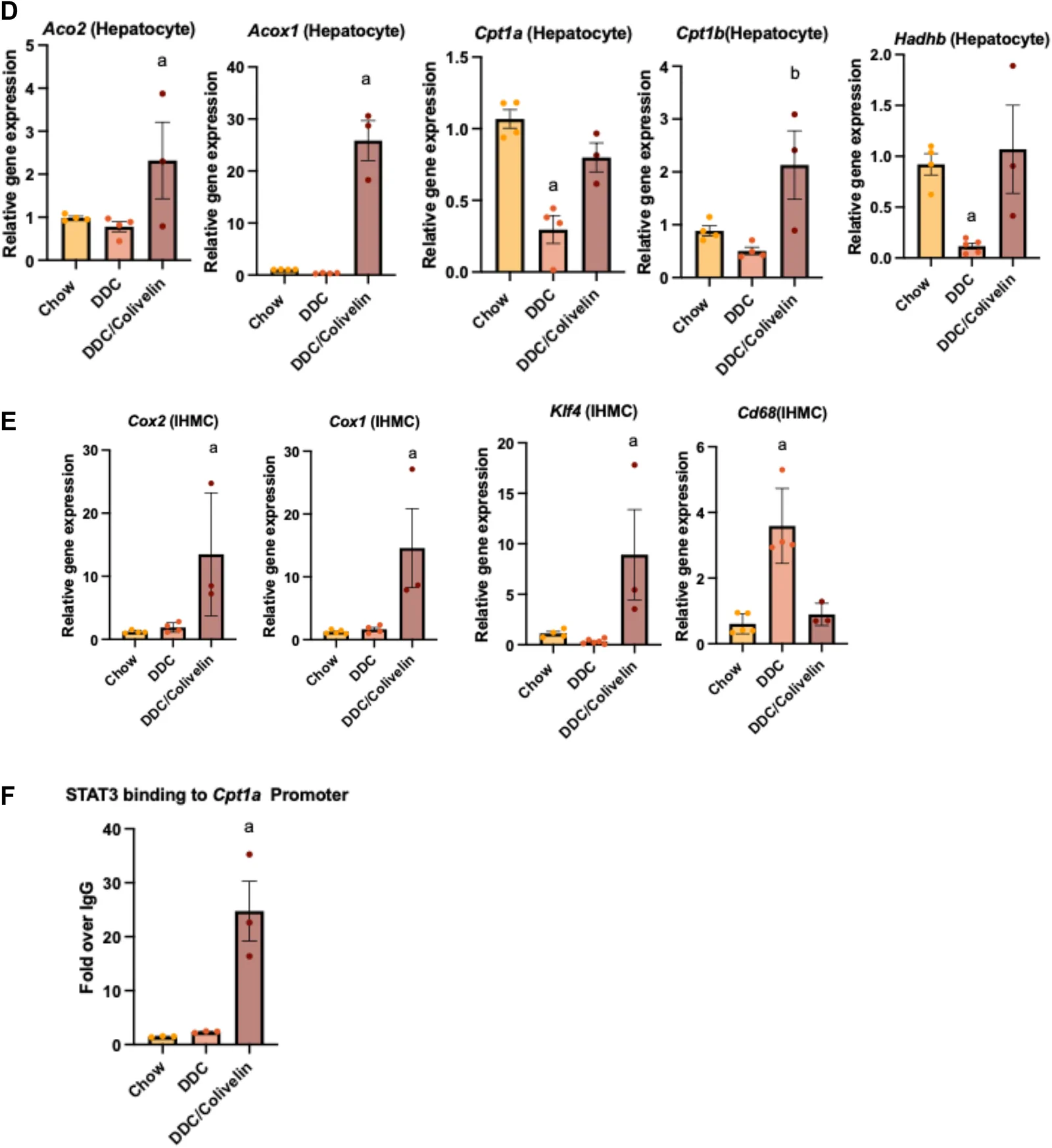
**Pharmacologic activation of STAT3 attenuates DDC-induced liver injury.***A*, serum liver injury markers show significant elevation in DDC mice. Serum total bilirubin, total bile acids, and alkaline phosphatase are significantly reduced in colivelin (STAT3 activator) treated DDC mice. *B*, representative H&E and immunohistochemistry and quantification showing increased F4/80 (100X) (macrophage marker) and CK19-(100X) (ductular marker) staining in liver sections from DDC mice with reduction of staining in DDC/colivelin treated mice. *C*, mRNA expression analysis by qPCR of genes involved in bile acid and bilirubin export (*Abcbb11*, *Nrob2*, *Abcc2*)) from hepatocytes isolated from Chow, DDC, and DDC/colivelin mice. *D*, mRNA expression analysis by qPCR of genes involved in fatty acid oxidation (*Aco2*, *Cpt1a*, *Acox1*, *Cpt1b*, *Hadhb*)) from hepatocytes isolated from Chow, DDC, and DDC/colivelin mice. *E*, mRNA expression analysis by qPCR of genes involved in AA metabolism (*Cox1*, *Cox2*) and anti-inflammatory (*Klf4*) and pro-inflammatory (*Cd68*) pathways from IHMCs isolated from Chow, DDC, and DDC/colivelin mice. *F*, ChIP analysis of STAT3 binding to the promoter of *Cpt1a* in liver from Chow, DDC, and DDC/colivelin mice. Data in (*A–F*) are presented as mean ± SEM (n = 3–5 mice per group). Statistical significance was determined using one-way ANOVA with appropriate multiple-comparison correction. a = *p* < 0.05 *versus* all other groups; b = *p* < 0.05 *versus* DDC mice.

To determine if STAT3 activation reverses AA-driven liver injury and inflammatory signaling, we examined inflammatory gene expression in cholangiocytes and macrophages. In cultured cholangiocyte-derived H69 cells, AA exposure overnight significantly increased the expression of inflammatory and proliferation mediators (*CCL2* and *KRT19*) while treatment with colivelin reduced these responses (Fig. S6*A*). Similarly, in THP-1 macrophages, AA induced the expression of *IL1B* and *CCL2*, which were suppressed following colivelin treatment. (Fig. S6*A*).

STAT3 activation in IHMCs isolated from DDC/colvelin mice also increased the expression of AA metabolism genes that are *Stat3* targets (*Cox1*, *Cox2*) and the anti-inflammatory gene *Klf4*, while it suppressed expression of the pro-inflammatory *Cd68*, suggesting a role for STAT3 in suppressing inflammation through AA-derived signaling pathways (Fig. 6*E*). Furthermore, ChIP assay showed increased binding of STAT3 to *Cpt1a* promoter in liver from DDC/colivelin mice compared to DDC and Chow mice (Fig. 6*F*). STAT3 activation in cultured BMDMs exposed to DDC also induced expression of anti-inflammatory genes (*Arg1* and *Clec10a1*: Fig. S6*B*). Furthermore, expression of fibrogenesis genes was suppressed by colivelin treatment in DDC mice (Fig. S6*C*).

Collectively, these findings show that STAT3 activation reduces cholestatic liver injury by decreasing inflammation and ductular reaction, restoring bile acid transport, and promoting metabolic reprogramming toward fatty acid oxidation.

### FXR-STAT3 signaling promotes fatty acid oxidation and metabolic reprogramming of hepatic macrophages during cholestatic injury

We next investigated whether FXR signaling influences macrophage metabolic reprogramming related to FAO. Gene expression analysis indicated that DDC-induced cholestatic injury greatly reduced the expression of FAO-related genes in liver tissues. Conversely, activating FXR signaling with GW4064 significantly increased the hepatic expression of key FAO regulators such as *Cpt1a*, *Cpt2*, *Cpt1b*, and *Hadhb* (Fig. 7*A*). Likewise, genes involved in lipid metabolism and mitochondrial β-oxidation, including *Acsf2 and Acox1*, were markedly upregulated following FXR activation (in DDC/GW4064 mice) compared to DDC mice. These results suggest that FXR signaling helps to restore hepatic FAO, which is suppressed during cholestatic injury. To further probe the role of STAT3 in this FXR effect, we performed co-treatment with the STAT3 inhibitor C-188-9 and show that it partially or completely attenuated this FXR response, indicating that STAT3 contributes to FXR-mediated reprogramming of FAO genes during cholestatic liver injury (Fig. 7*A*).

**Figure 7:**
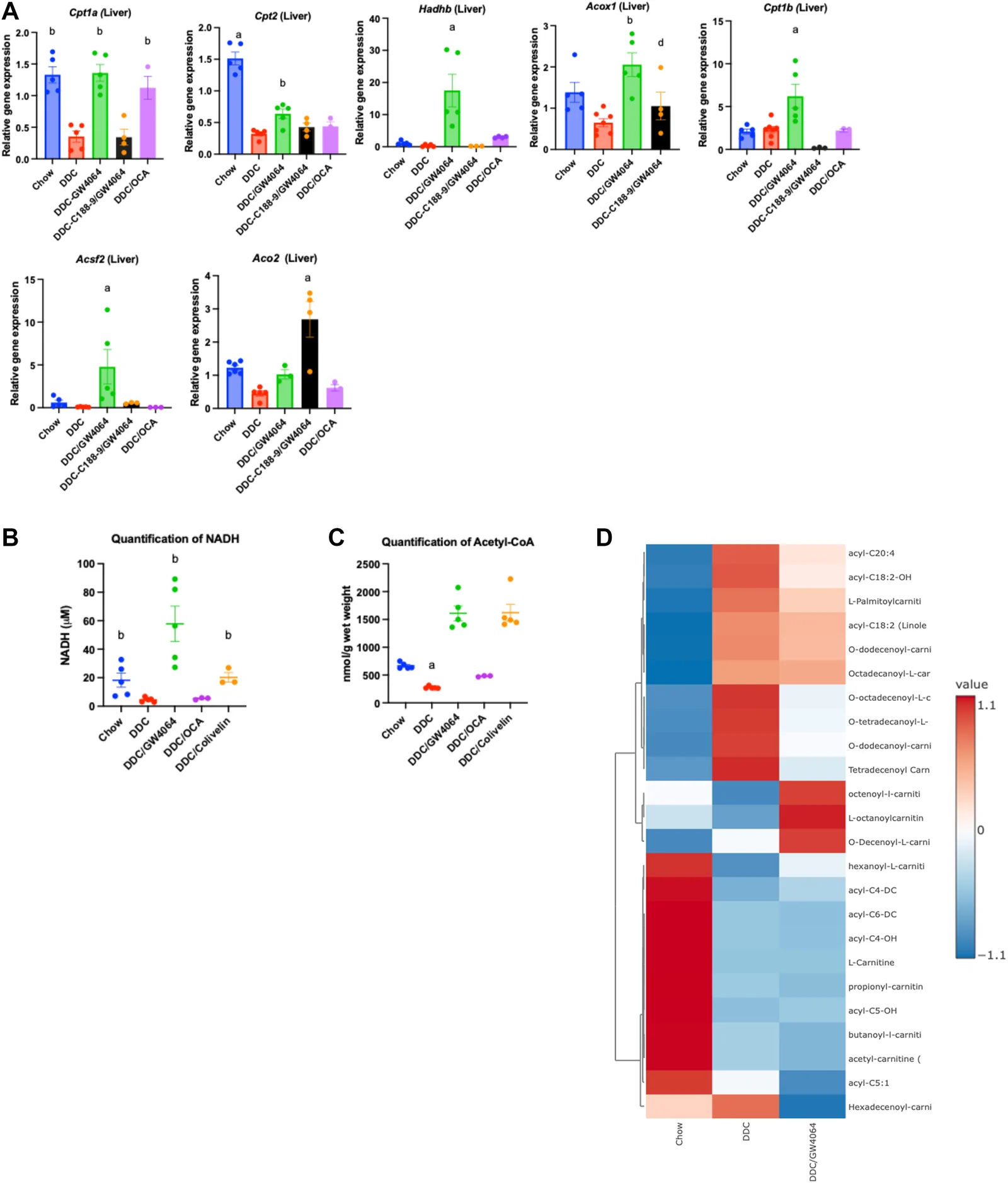

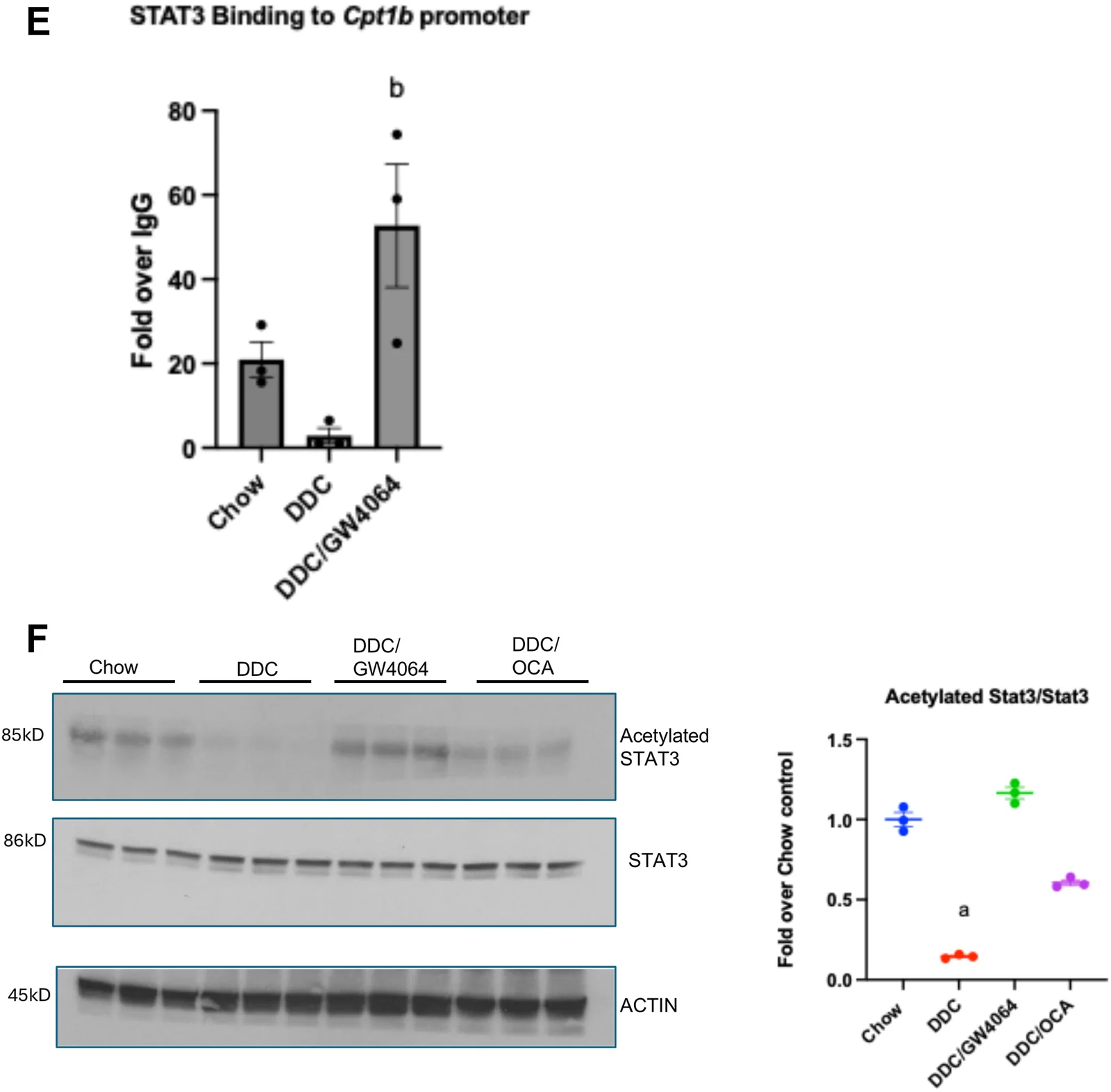

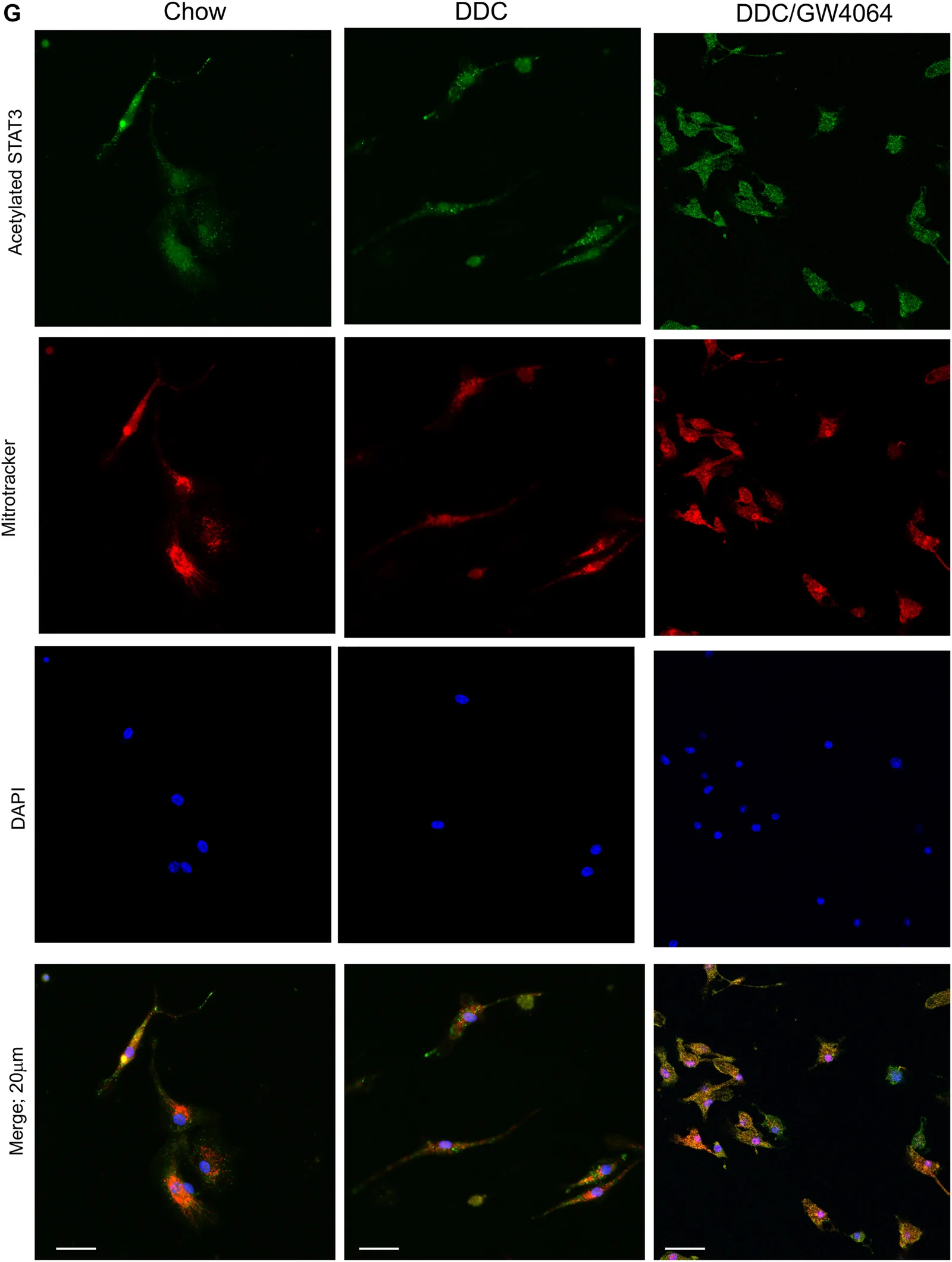

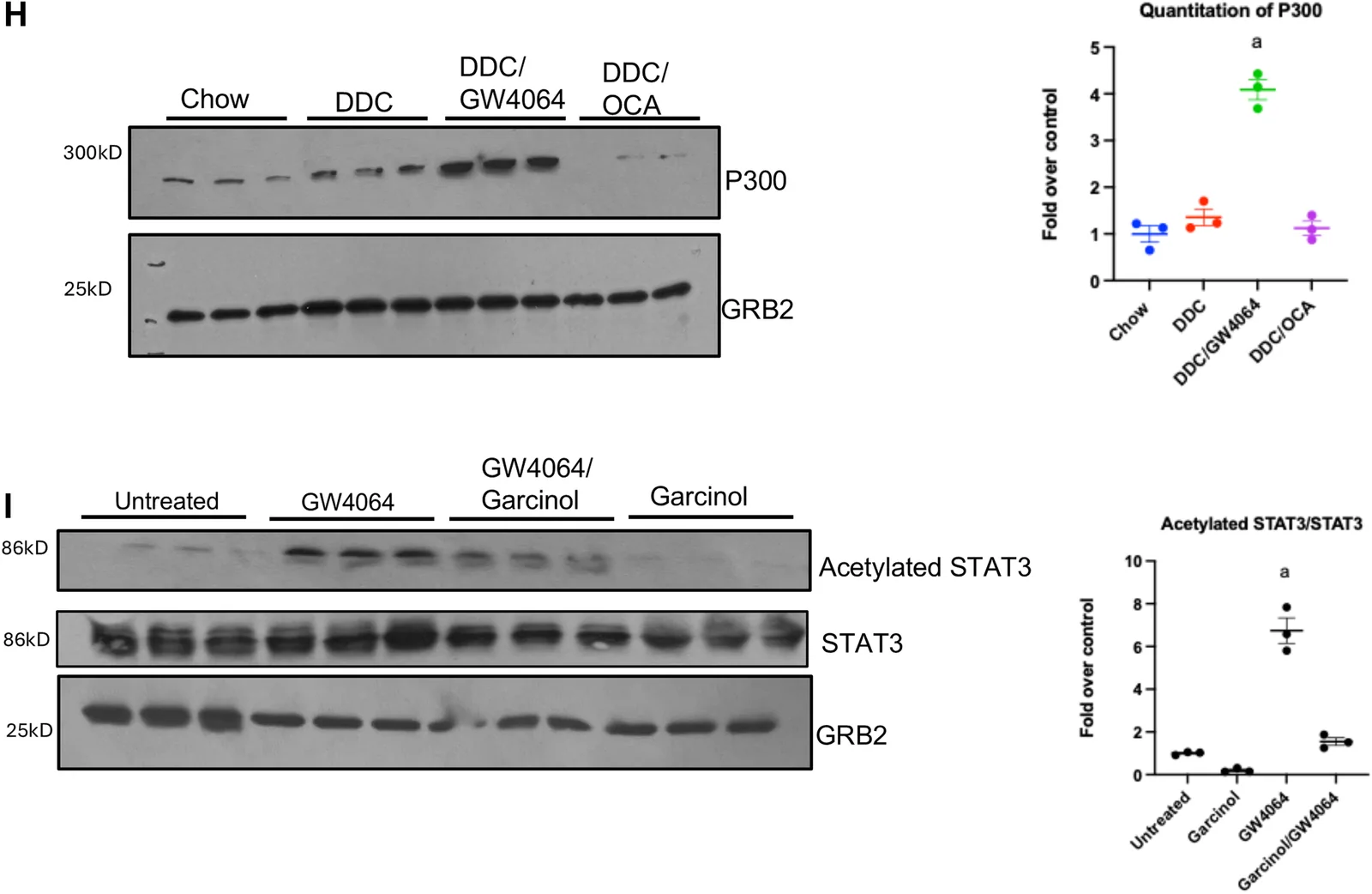

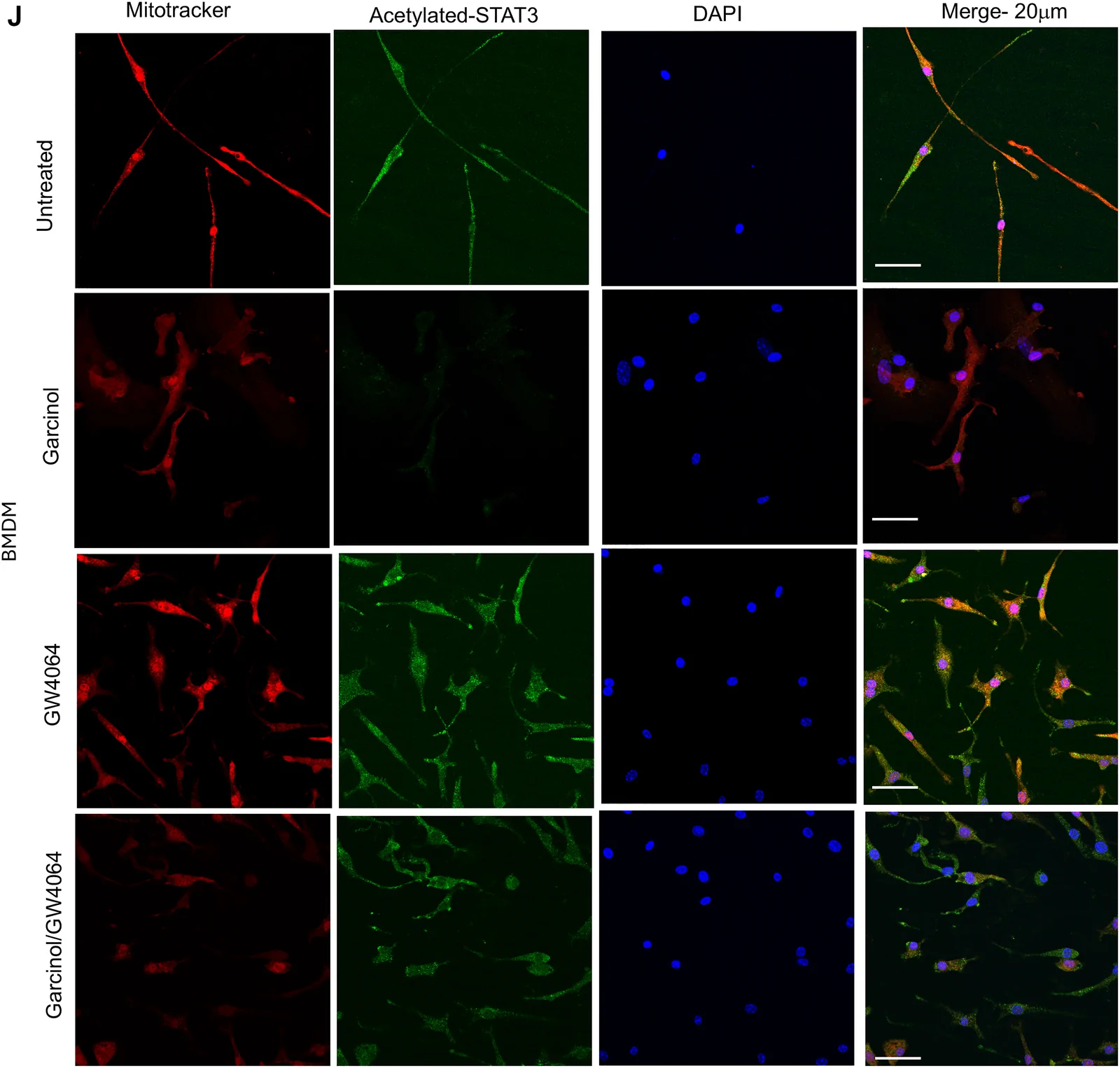

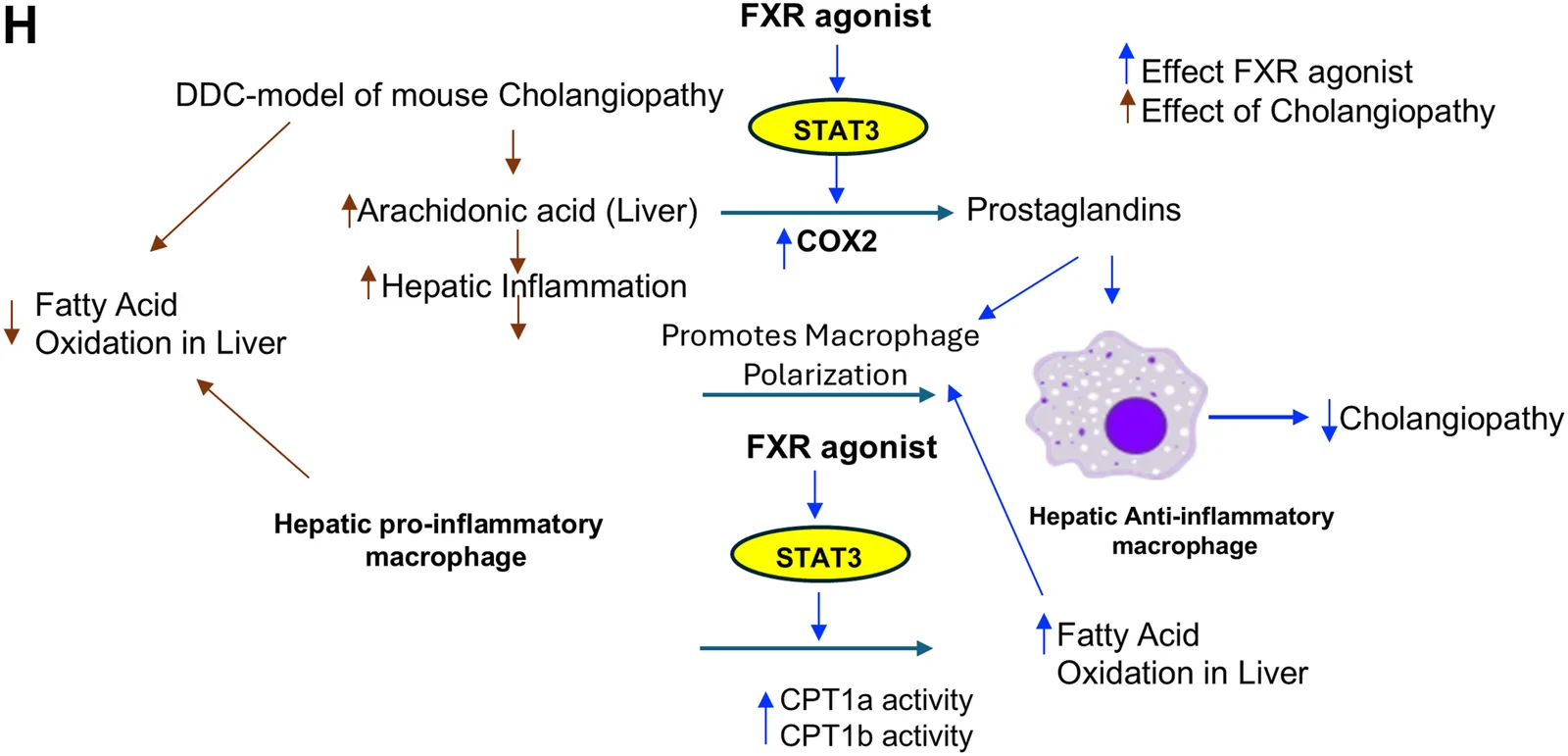
**FXR–STAT3 signaling promotes molecular regulation of fatty acid oxidation and metabolic reprogramming during cholestatic liver injury.***A*, gene expression analysis of fatty acid oxidation genes (*Cpt1a*, *Cpt2*, *Cpt1b*, *Hadhb*, *Actb*, *Acsf2*, *Acox1*, *Acox2*) in liver tissues from Chow, DDC, DDC/GW4064, and DDC/OCA mouse groups, showing upregulation of all genes by GW4064 during DDC cholangiopathy. Co-treatment with the STAT3 inhibitor C-188-9 attenuated these protective effects in DDC mice treated with GW4064. *B* and *C*, measurement of NADH and acetyl-CoA levels in liver homogenate showing reduced levels in DDC and enhanced levels in DDC/GW4064 and DDC/Colivelin mice. *D*, metabolomics analysis of portal serum from Chow, DDC, and DDC/GW4064 mouse groups displayed as heatmaps shows differential abundance of FAO metabolites (Carnitine) across experimental groups. *E*, ChIP analysis of mouse liver shows reduced STAT3 binding to the *Cpt1b* promoter in DDC mice and enhanced STAT3 binding to the *Cpt1b* promoter in DDC/GW4064 mice. Representative images of three biological replicates are shown. *F*, Western blot analysis of acetylated STAT3 and total STAT3 in liver tissues and quantification of the acetylated STAT3/STAT3 ratio, demonstrating suppression in DDC mice and normalized STAT3 acetylation by GW4064 treatment. The total STAT3 and ACTIN blots shown in Figure 7*F* are derived from the same experimental blot shown in Figure 5*C* and are reproduced here because the corresponding samples were analyzed for both phosphorylated and acetylated STAT3. *G*, immunofluorescence confocal analysis of acetylated STAT3 localization in BMDMs from 14-days treated mice, showing co-localization of STAT3 with Mitotracker red in mitochondria of DDC/GW4064 mouse cells. *H*, Western blot analysis of p300 in liver tissues of Chow, DDC, DDC/GW4064, and DDC/OCA and quantification, demonstrating that GW4064 treatment increased P300 protein in DDC mice. *I*, Western blot analysis and quantitation of acetylated STAT3 in Raw264.7 cells with indicated treatments, demonstrating acetylated STAT3 induction by GW4064 that was suppressed by garcinol, an inhibitor of p300. *J*, immunofluorescence confocal analysis of acetylated STAT3 and its co-localization within mitochondria in BMDMs from untreated and garcinol treated in the presence or absence of GW4064. Untreated cells show acetylated STAT3 that is not co-localized with mitotracker. Garcinol treated cells show reduction in acetylated STAT3 and no colocalization. GW4064-treated cells show acetylation STAT3 colocalization in mitochondria and partially restored co-localization in Garcinol-treated cells. Data in (*A–C*, *E*, *F*, *H* and *I*) are presented as mean ± SEM (n = 3–5 mice per group). Statistical significance was determined using one-way ANOVA with appropriate multiple-comparison correction. a = *p* < 0.05 *versus* all other groups; b = *p* < 0.05 *versus* DDC mice. *K*, proposed mechanism of FXR-STAT3 mediated immunometabolic reprogramming during cholestatic liver injury. Activation of FXR promotes STAT3 phosphorylation and acetylation and mitochondrial translocation, driving metabolic reprogramming of hepatic macrophages. STAT3 activation improves fatty acid oxidation and arachidonic acid (AA) metabolism while suppressing pro-inflammatory gene expression, resulting in a shift from pro-inflammatory to anti-inflammatory macrophages. These changes decrease hepatic inflammation, ductular injury, and fibrosis, thereby promoting the resolution of cholestatic liver injury.

In parallel with these transcriptional changes, hepatic levels of NADH were significantly increased following GW4064 and colivelin treatment (Fig. 7*B*). This elevation suggests enhanced reducing equivalent generation, likely reflecting increased flux β-oxidation, and the tricarboxylic acid (TCA) cycle (18). Consistent with this metabolic shift, levels of acetyl-CoA were increased (Fig. 7*C*), indicating augmented substrate oxidation and mitochondrial acetyl-CoA production. The coordinated rise in NADH and acetyl-CoA suggests that FXR-mediated STAT3 activation promotes oxidative metabolic pathways.

Heatmap analysis of long-chain acylcarnitine species demonstrated distinct metabolic remodeling across experimental groups. Compared to Chow controls, DDC-treated samples exhibited altered levels of multiple long-chain acylcarnitines, including L-palmitoylcarnitine (C16), linoleoyl-carnitine (C18:2), octadecanoyl-carnitine (C18) and acyl-C20:4 (a long-chain acylcarnitine derived from AA, consistent with impaired mitochondrial fatty acid β-oxidation). FXR activation by GW4064 resulted in a marked increase in specific long-chain acylcarnitines, particularly unsaturated species, while some saturated intermediates showed partial normalization (Fig. 7*D*). This pattern suggests enhanced fatty acid flux and incomplete β-oxidation, indicative of metabolic reprogramming rather than full restoration of oxidative capacity.

ChIP analysis of liver homogenate demonstrated reduced STAT3 binding at the *Cpt1b* promoter in liver from DDC mice, which was markedly enhanced in DDC/GW4064 mice (Fig. 5*E*). Since previous results indicated that FXR influences STAT3 signaling, we next examined STAT3 post-translational modifications during cholestatic injury. Western blot analysis showed that acetylated STAT3 (Lys 87) levels were significantly lower in DDC mouse liver compared to Chow controls, while treatment with GW4064 restored STAT3 acetylation (Fig. 7*F*). Complementary transcriptomic analyses of mouse liver further supported these findings, showing large clusters of genes involved in FAO and metabolic pathways that were suppressed in livers from DDC mice (Fig. S7*A*) and were restored following FXR agonist treatment (Fig. S7*B*). To determine whether STAT3 directly regulated FAO-related genes, we examined the effect of FXR activation on acetylated STAT3 localization in mitochondria. Immunofluorescence analysis of BMDMs isolated from experimental mice demonstrated enhanced acetylated STAT3 mitochondrial co-localization by FXR activation in DDC/GW4064 mice. (Fig. 7*G*). To identify the upstream mechanism driving STAT3 acetylation, we assessed expression of the histone acetyltransferase p300. Western blot analysis revealed a marked increase in p300 protein levels in GW4064-treated liver compared to control and DDC groups, supporting a role for p300 in mediating FXR-induced STAT3 acetylation (Fig. 7*H*). To further evaluate whether p300 contributes to FXR-induced STAT3 acetylation, RAW264.7 macrophages were treated with the p300 acetyltransferase inhibitor garcinol in the presence of GW4064. FXR agonist treatment increased STAT3 acetylation, whereas garcinol significantly attenuated GW4064-induced STAT3 acetylation as determined by Western blot analysis (Fig. 7*I*, upper panels). Consistent with these findings, immunofluorescence staining demonstrated that garcinol markedly reduced FXR-induced acetylated STAT3 in BMDM macrophages (Fig. 7*J*). These findings support a functional role for p300 activity in mediating STAT3 acetylation downstream of FXR activation.

We next examined P300 induction of STAT3 acetylation in mitochondria of Raw 264.7 macrophages. First, we show by immunoblotting in Raw 264.7 macrophages treated with FAO byproduct acetyl-CoA that acetyl Co-A increased the P300 protein (Fig. S7*C*). Next, following acetyl-CoA treatment overnight in Raw 264.7 cells, we show by confocal microscopy the presence of strong colocalization of p300 within mitochondria (Fig. S7*D*, upper panel). Finally, acetyl-CoA treatment overnight in Raw 264.7 cells demonstrated increased acetylated STAT3. (Fig. S7*E*, lower panel).

Together, these findings demonstrate that FXR activation induces STAT3 acetylation and transcriptional activity to promote FAO and metabolic reprogramming in hepatic macrophages. This mechanism offers an essential link between FXR signaling, lipid mediator metabolism, and macrophage polarization during cholestatic liver injury.

## Discussion

Chronic cholestatic liver diseases, including inflammatory cholangiopathies, are marked by perturbations of bile acid homeostasis, hepatic immune activation, and ongoing bile ductular injury (19). In this study, we identify a mechanistic pathway connecting FXR signaling, STAT3 activation, macrophage immunometabolism, and AA–driven inflammatory signaling in cholestatic liver injury (8). Our results show that activating FXR may reduce inflammatory macrophage polarization, enhance fatty acid oxidative metabolism, and ultimately slow the progression of cholangiopathy in the DDC mouse model of intrahepatic cholestasis. These findings provide a mechanistic framework in which FXR–STAT3 signaling integrates metabolic and immune responses to protect against cholestatic injury. It has been shown that FXR may protect against cholestatic liver injury through transcriptional regulation of bile acid synthesis and transport genes in experimental models of cholestasis (5, 20, 21, 22). Consistent with these findings, our data demonstrate that treatment with FXR agonists in the DDC mouse model attenuates cholestatic liver injury in conjunction with reducing macrophage infiltration and activation, restoring bile acid metabolic homeostasis and fatty acid oxidation, and suppressing pro-fibrotic signaling pathways.

An important mechanistic insight from our study is the role of STAT3 as a key mediator of FXR-dependent hepatoprotection (6, 8). Previous studies have demonstrated that STAT3 signaling contributes to hepatocyte survival and anti-inflammatory responses during liver injury (23). Furthermore, recent work from our laboratory has shown that FXR agonists may mediate hepatoprotective effects in parenteral nutrition-associated cholestasis through induction of STAT3 signaling (8). Our current findings expand on these observations by showing that FXR activation promotes STAT3 phosphorylation and acetylation, increases STAT3 binding to metabolic (*e*.*g*., FAO) gene promoters, and regulates transcriptional programs linked to fatty acid oxidation. These results suggest that FXR-dependent STAT3 activation acts as a critical molecular switch in connecting metabolic signaling with inflammatory regulation during cholestatic injury.

Our ChIP analyses demonstrated enhanced FXR and STAT3 occupancy at target promoters following FXR activation. This increased promoter binding may coincide with enhanced STAT3 acetylation at Lys87 and STAT3 Tyr705 phosphorylation, suggesting that post-translational modifications of STAT3 contributes to promotor binding and increasing transcription of its target genes. In addition, GW4064 treatment of DDC mice enhanced JAK2 and STAT3 Tyr705 phosphorylation, suggesting that GW4064 may activate upstream JAK2 phosphorylation of STAT3. Although the present study does not define the complete molecular mechanism regulating promoter binding, these findings support a model in which FXR-induced STAT3 activation facilitates transcriptional regulation of its downstream genes involved in FAO and macrophage immunometabolic reprogramming.

A major finding of this study is that FXR signaling regulates macrophage immunometabolism and FAO pathways. Hepatic macrophages are key regulators of cholestatic inflammation and fibrosis, integrating signals from bile acids, cytokines, and lipid mediators (24, 25, 26). Our data show that FXR activation suppresses expression of pro-inflammatory macrophage genes while promoting anti-inflammatory macrophage markers. These findings are consistent with previous studies demonstrating that FXR stimulation promotes anti-inflammatory macrophage polarization and anti-inflammatory responses (27, 28, 29, 30). In the current study, we now demonstrate that FXR-STAT3 signaling induces expression of genes involved in mitochondrial FAO, including *Cpt1a*, *Cpt1b*, *and Acox1*, demonstrating the association between metabolic reprogramming toward oxidative metabolism with anti-inflammatory macrophage phenotype polarization.

It should be noted that STAT3 inhibition with C-188-9 partially reversed the protective effects of GW4064 on cholestatic injury, macrophage accumulation, ductular reaction, and fibrosis. C-188-9 also attenuated FXR-mediated induction of FAO genes, supporting STAT3 as a functional mediator of FXR-dependent immunometabolic reprogramming. The incomplete reversal suggests that additional STAT3-independent FXR pathways also contribute to hepatoprotection.

This study also demonstrates that AA-derived lipid mediators likely play a role in inflammatory signaling during cholestatic injury. AA metabolism produces prostaglandins that control anti-inflammatory responses and immune cell activation (31). Elevated metabolites from AA have been linked to metabolic dysfunction-associated steatotic liver disease (32); however, no association with cholestasis has been reported. In this study, lipidomic analyses indicated significant changes in oxylipin metabolites during cholestatic injury (33). AA biology is highly context-dependent. Although AA supplementation can be protective in some models, our data indicate that AA-associated oxylipin remodeling promotes inflammatory signaling during DDC-induced cholangiopathy. Importantly, activating FXR suppressed AA-induced inflammatory gene expression and decreased macrophage activation, suggesting that FXR signaling counters lipid mediator-driven inflammatory pathways. Beyond inflammation, our findings indicate that FXR signaling also suppresses TGF-β-driven fibrotic remodeling, a key feature of progressive cholangiopathies. Activation of TGF-β signaling promotes extracellular matrix deposition and ductular proliferation during chronic liver injury (34, 35). We observed that FXR agonist treatment significantly reduced expression of pro-fibrotic genes, including *Spp1*, *S100a4*, *Mmp13*, *and Col1a1* and reduced histologic ductular reaction and portal expansion (36). These findings suggest that metabolic reprogramming of hepatic macrophages may limit fibrotic remodeling through reduced inflammatory signaling and cytokine production.

Pharmacologic activation of STAT3 with colivelin in DDC mice decreased inflammatory gene expression in hepatocytes and macrophages, lowered macrophage accumulation, and lessened cholestatic injury. These results support the concept that STAT3 acts as a central downstream mediator of FXR signaling (23, 37). Mechanistically, STAT3 drives macrophage immunometabolic reprogramming (38) by shifting hepatic macrophages from a pro-inflammatory to an anti-inflammatory, pro-resolving phenotype, coupled with enhancement of fatty acid oxidation and anti-inflammatory lipid mediator production. Consistently, the accumulation of arachidonoyl-carnitine following FXR activation indicates increased AA utilization, linking mitochondrial fatty acid flux to oxylipin-mediated immunoregulatory signaling (32, 39). Notably, FXR activation also increased hepatic p300 levels and STAT3 acetylation and translocation to mitochondria, suggesting that p300-mediated STAT3 acetylation facilitates mitochondrial translocation of STAT3 (38). These data support a p300-dependent mechanism for STAT3 acetylation downstream of FXR signaling, although direct enzymatic acetylation remains to be formally established in future studies. Once inside the mitochondria, the STAT3 likely regulates electron transport chain function (40) and redox balance, creating a mismatch between fatty acid influx and oxidative capacity, leading to accumulation of long-chain acylcarnitines and diversion of lipid substrates toward immunoregulatory pathways. Collectively, these findings identify STAT3-dependent metabolic reprogramming as a promising therapeutic target for resolving inflammation and promoting repair in cholestatic liver diseases.

One of the limitations of this study is that it did not employ hepatocyte- or macrophage-specific FXR or STAT3 knockout mice to determine the relative role of these cells in the effect of FXR-STAT3 signaling in this model. However, the combined use of pharmacologic STAT3 activation and inhibition *in vivo* together with STAT3 siRNA-mediated knockdown in hepatocyte and macrophage cell systems provide complementary evidence supporting STAT3 as a functional mediator of FXR action during cholestatic injury. Nevertheless, because FXR is expressed in multiple hepatic cell populations, future studies using cell type-specific genetic models will be required to determine the relative contributions of hepatocyte- and macrophage-specific FXR-STAT3 signaling and the importance of intercellular cross-talk in mediating hepatoprotection.

In conclusion, this study extends our previous observations of FXR-STAT3 signaling by identifying a previously unrecognized immunometabolic mechanism through which STAT3 coordinates mitochondrial FAO, arachidonic acid metabolism, and macrophage polarization during cholestatic liver injury. These findings provide mechanistic insight into how FXR integrates metabolic and immune pathways to limit inflammation and fibrosis in addition to its effects on bile acid metabolism and suggest that therapeutic targeting of the FXR-STAT3 immunometabolic axis may characterize a promising approach for PSC and other inflammatory cholangiopathies. The potential therapeutic benefits of FXR activation may extend beyond bile acid regulation to include modulation of macrophage metabolism and inflammatory signaling pathways.

## Experimental procedures

### Mouse study

Male C57BL/6J mice (8 weeks) were fed 0.1% DDC for 14 days and treated with GW4064 (30 mg/kg/day), OCA (20 mg/kg/day), colivelin (10 mg/kg/day; i.v.), C-188-9 (10 mg/kg/day; i.p.), or AA (50 mg/kg/day; i.p.) under IACUC-approved conditions. Mice were randomized into Chow, DDC, and treatment groups, with interventions delivered *via* diet, intravenous infusion, or intraperitoneal injection. Blood, liver, and bone marrow were collected for serum biochemistry, metabolomics, histology, and BMDM generation. Serum ALT, AST, ALP, bilirubin, and total bile acids were quantified in a blinded manner using clinical chemistry assays. All procedures followed protocols approved by the Institutional Animal Care and Use Committee (IACUC) and adhered to ARRIVE guidelines. More complete details for all Methods are found in the Supplementary Methods.

### Intrahepatic mononuclear cells (IHMC) and hepatocyte isolation

IHMC were isolated by enzymatic digestion and density gradient centrifugation following liver perfusion. Primary hepatocytes were obtained using the collagenase perfusion method and purified by low-speed centrifugation for downstream analyses.

### siRNA knockdown and treatment

Raw264.7 and Huh7 cells were transfected with STAT3 siRNA or control siRNA and treated with AA (50 μM) ± GW4064 (5 μM). Cells were processed for downstream molecular analyses.

### Immunoblot, immunofluorescence, ChIP assay, and histological analysis

Protein expression was assessed by SDS-PAGE and immunoblotting using specific antibodies and ECL detection. Cells were stained for acetyl-STAT3, P300, and pSTAT3 and imaged by confocal microscopy. ChIP was performed to assess FXR and STAT3 binding at target gene promoters.

Liver sections were analyzed by H&E and immunohistochemistry for F4/80 and CK19.

### ELISA and caspase activity assay

Serum IL6 levels were quantified using a commercial ELISA kit following the manufacturer’s instructions. Caspase-8 and -3/7 activities were measured using luminescent Caspase-Glo assays.

### qPCR and RNA-seq

Gene expression was quantified by TaqMan-based qRT-PCR and normalized to internal controls. RNA-seq libraries were generated and analyzed for differential expression (DESeq2, FDR <0.05) and pathway enrichment (GSEA).

### Co-culture experiments

H69 cholangiocytes and THP-1 cells were co-cultured using transwell systems to assess FXR-STAT3 signaling.

### Metabolomics and oxylipin analysis

Metabolites and oxylipins were extracted from mouse liver and portal serum and analyzed by UHPLC–HRMS using targeted and untargeted approaches. Data processing, annotation, and quality control were performed using standard pipelines and internal standards.

### Annexin V apoptosis assay and CD95 flow cytometry

Hepatocyte apoptosis was quantified by Annexin V/PI staining followed by flow cytometry. Apoptotic CD95 (Fas) expression was measured using flow cytometry.

### Statistical analysis

Data are presented as mean ± SEM with statistical significance determined by *t* test or ANOVA with multiple comparison correction. Analyses were performed and coded using GraphPad Prism and R with blinded data acquisition. Detailed protocols for all methods and statistical analyses are provided in the Supplementary Methods.

### Data availability

Data will be made available on reasonable request.

## Supporting information

This article contains supporting information.

## Conflict of interest

The authors declare the following financial interests/personal relationships which may be considered as potential competing interests: R. J. S. consults for Ipsen/Albireo, Mirum, Mallinckrodt, Astellas, and ProQR; these companies had no role in this project.

## Figures and Tables

**Fig. S1:**
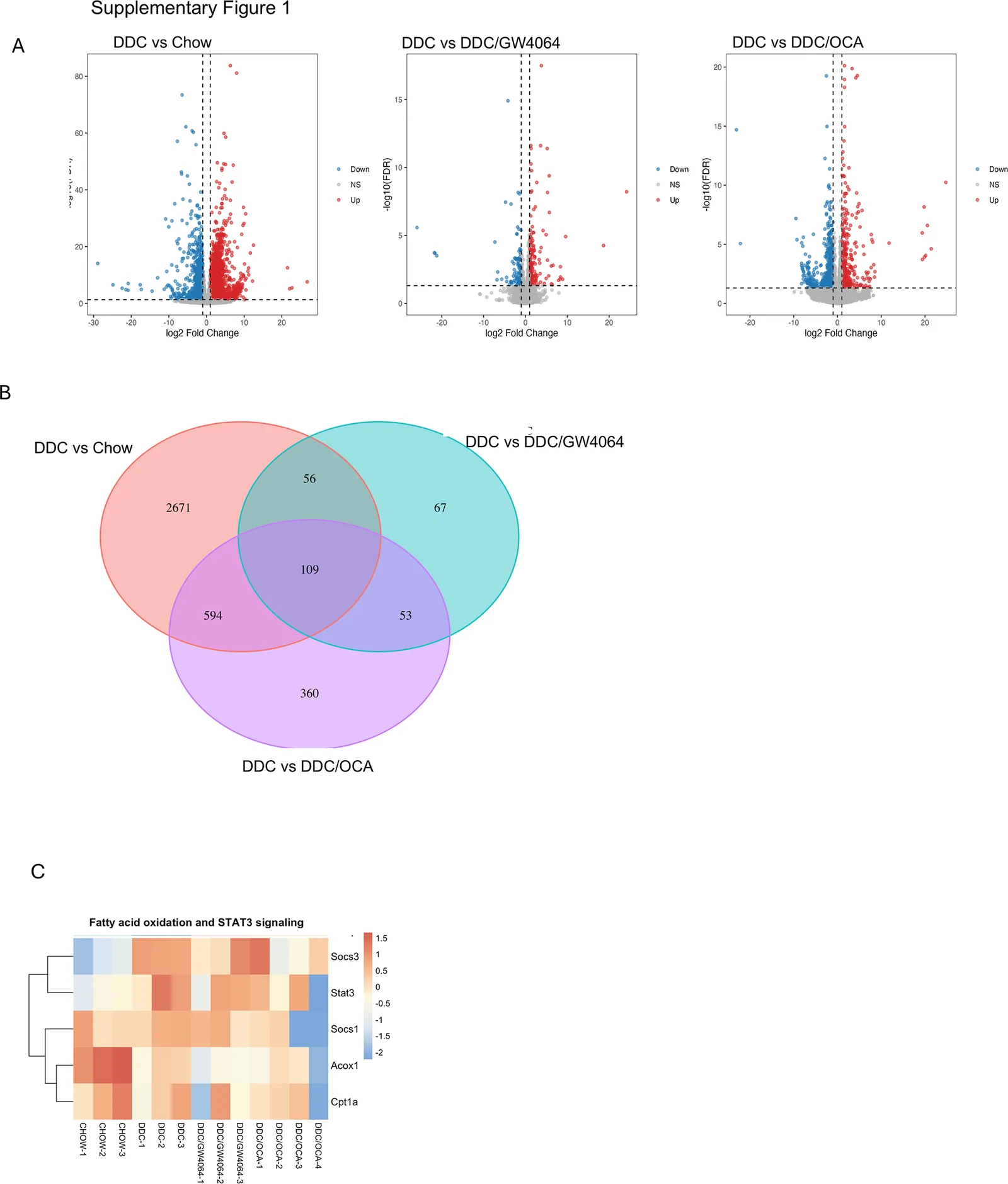


**Fig. S2.1:**
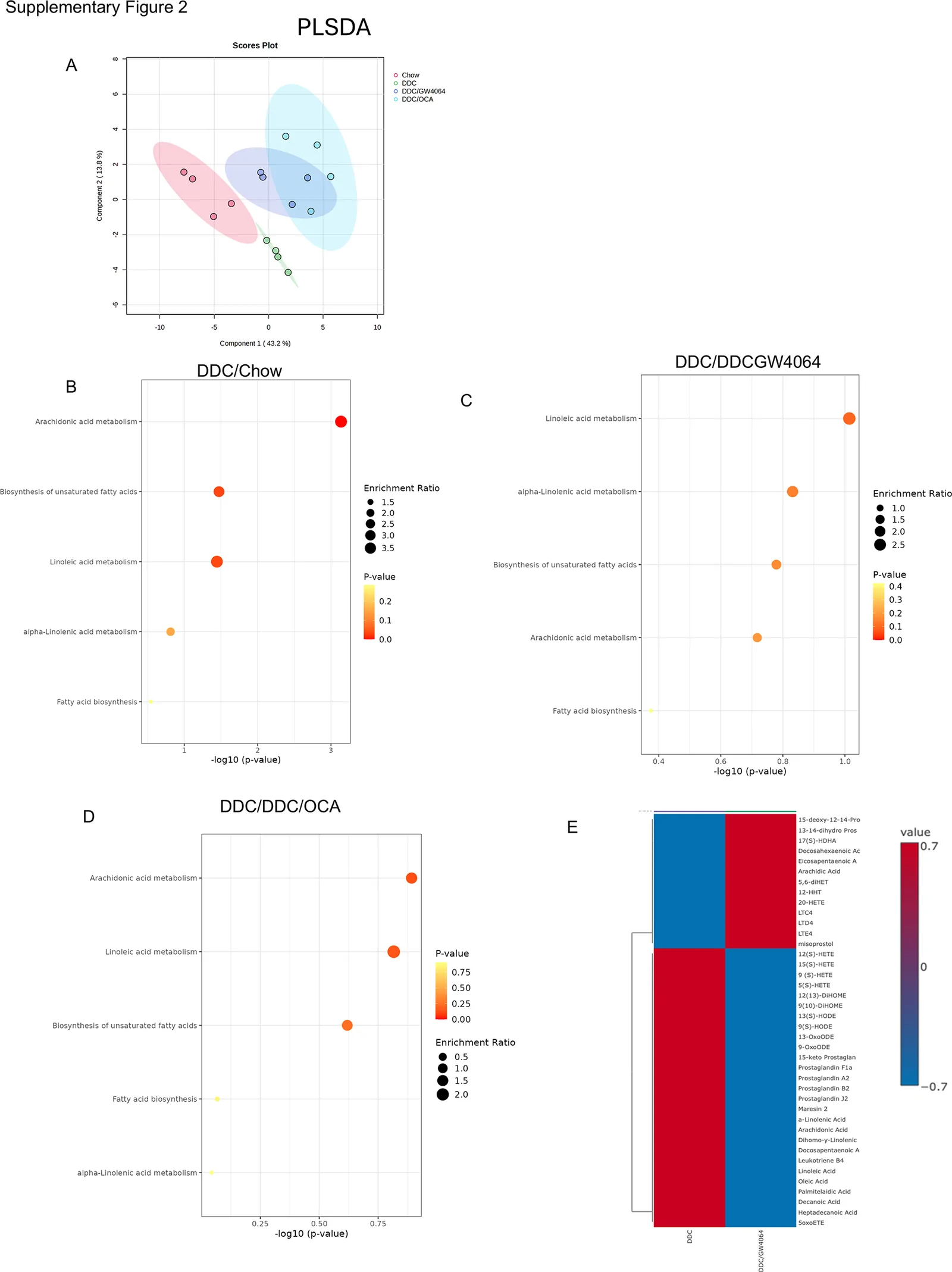


**Fig. S2.2:**
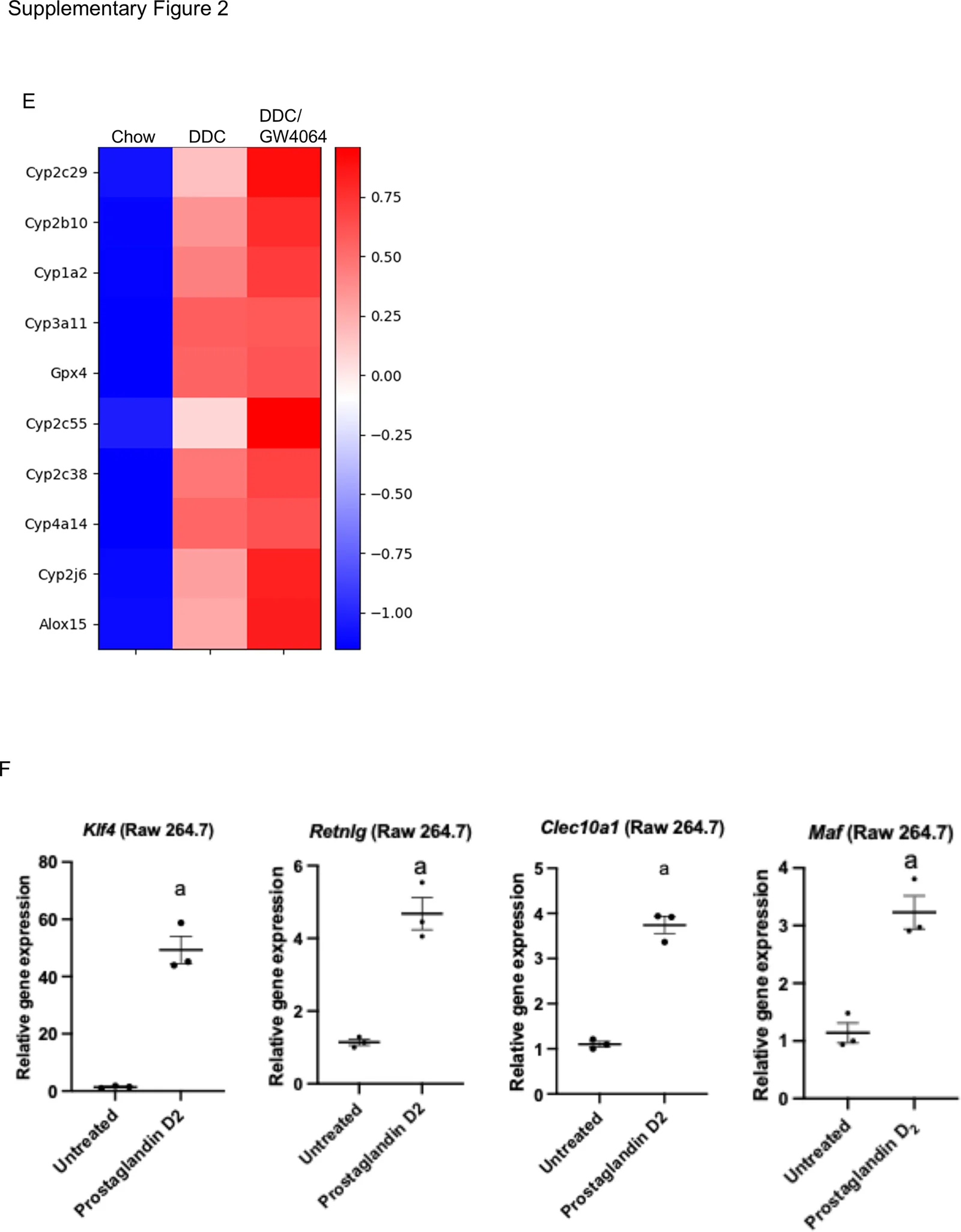


**Fig. S3.1:**
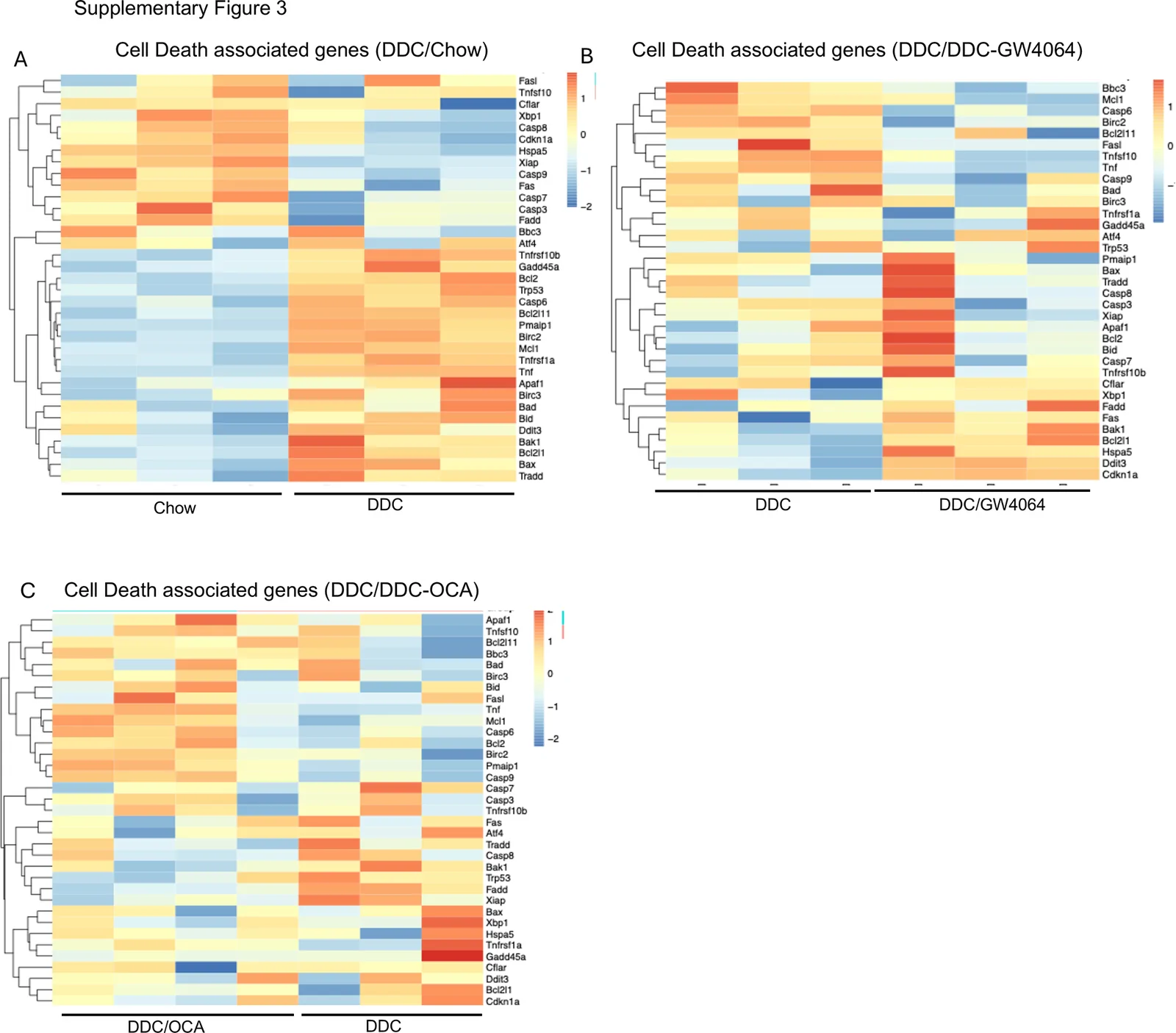


**Fig. S3.2:**
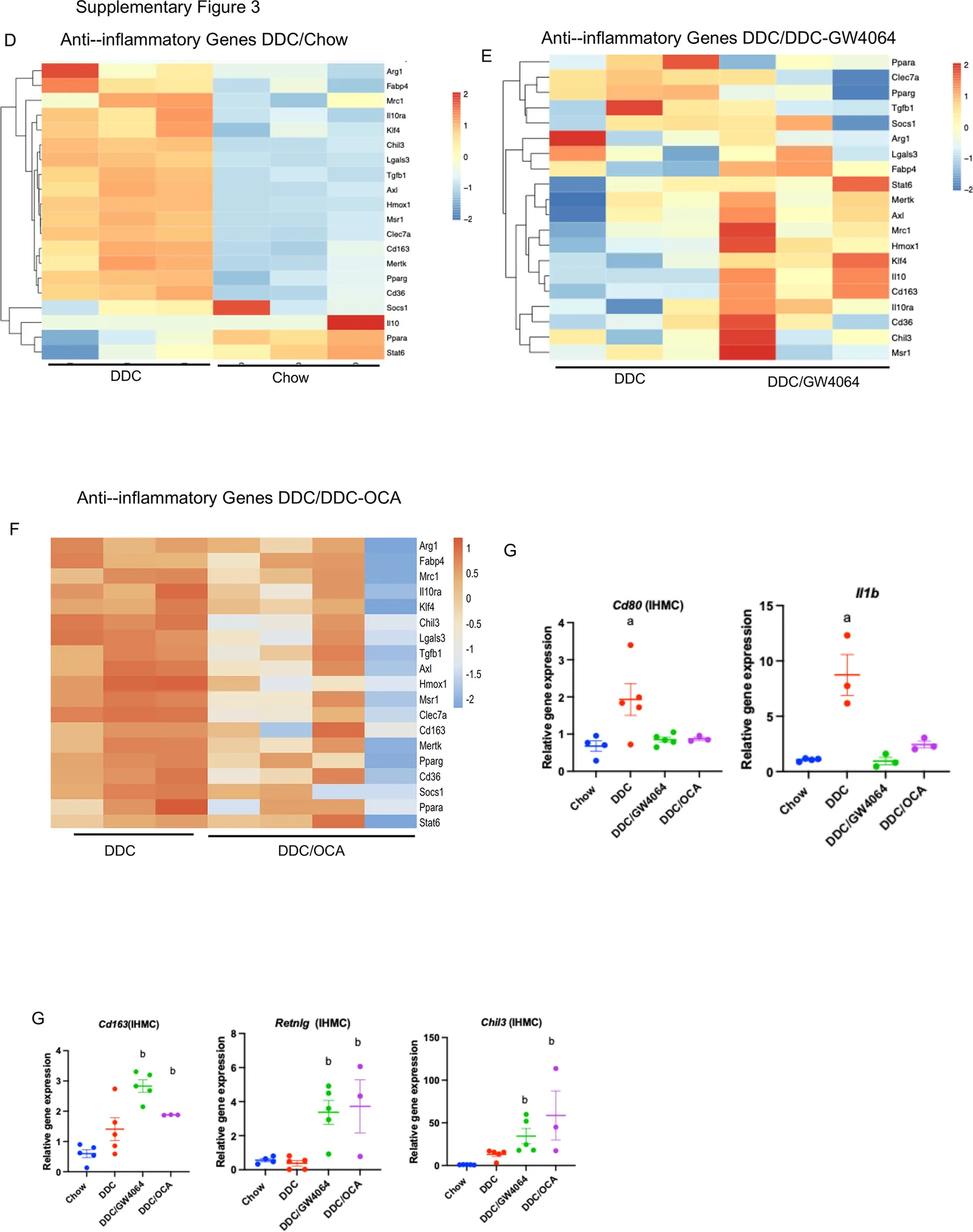


**Fig. S4:**
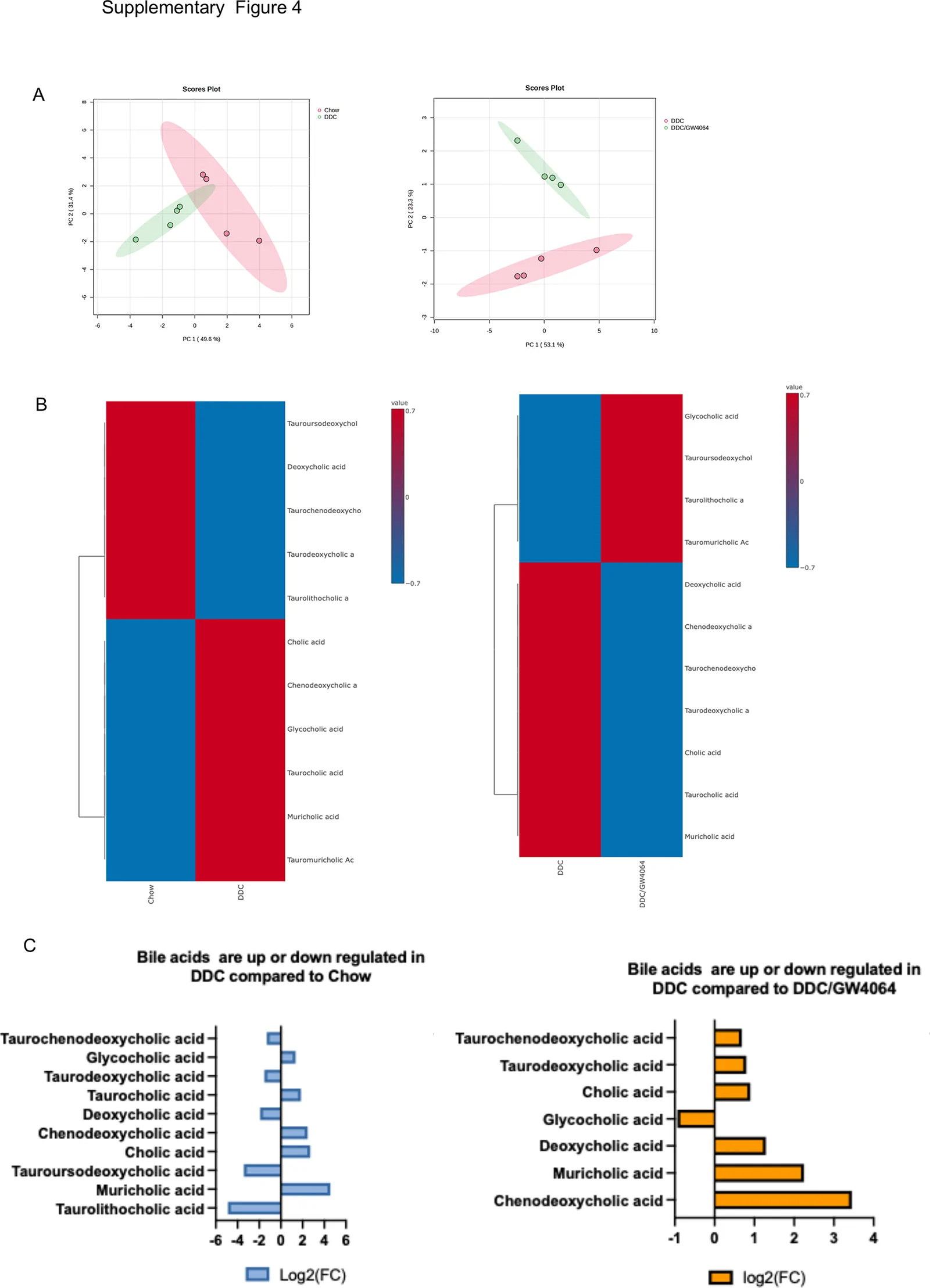


**Fig. S5:**
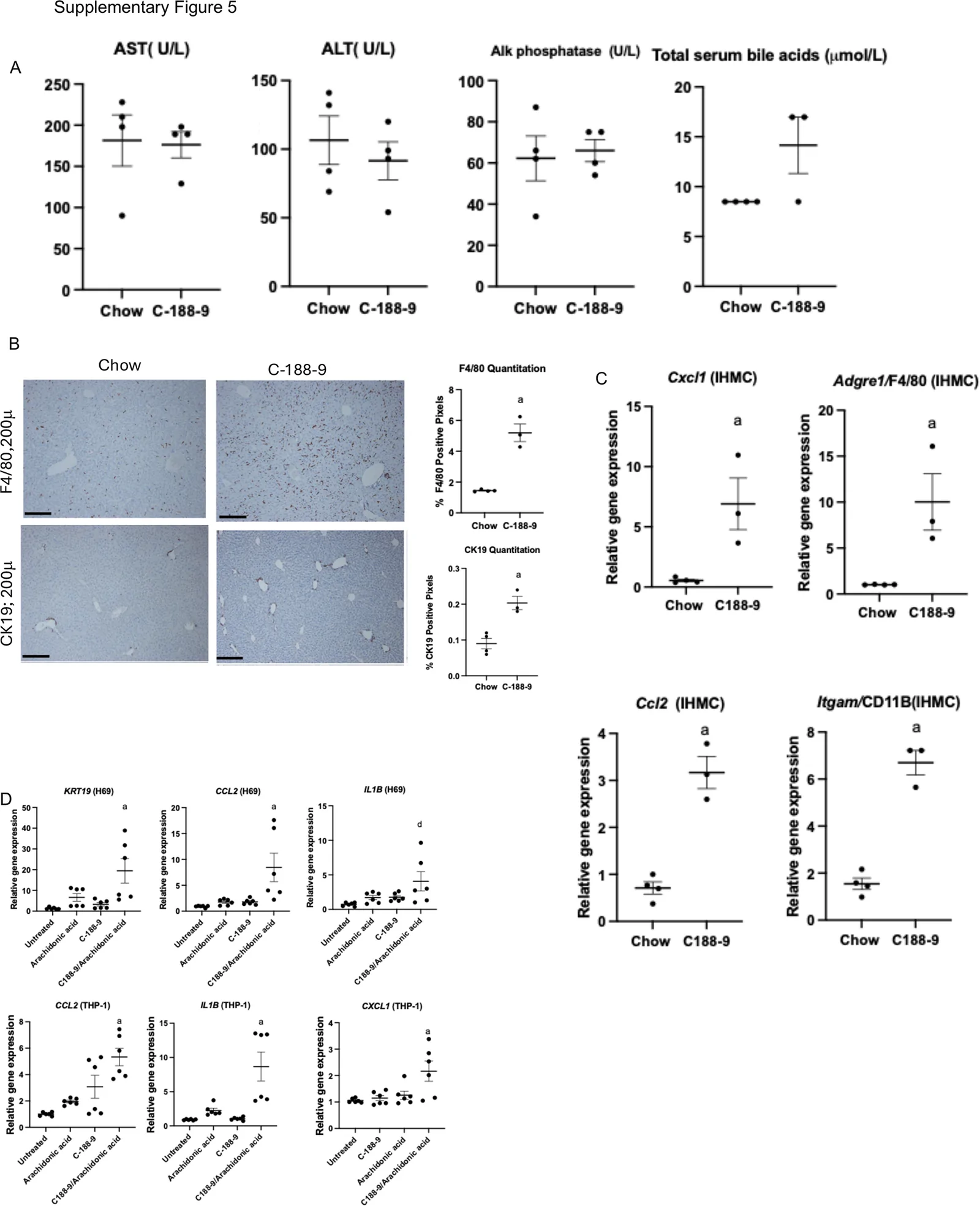


**Fig. S6:**
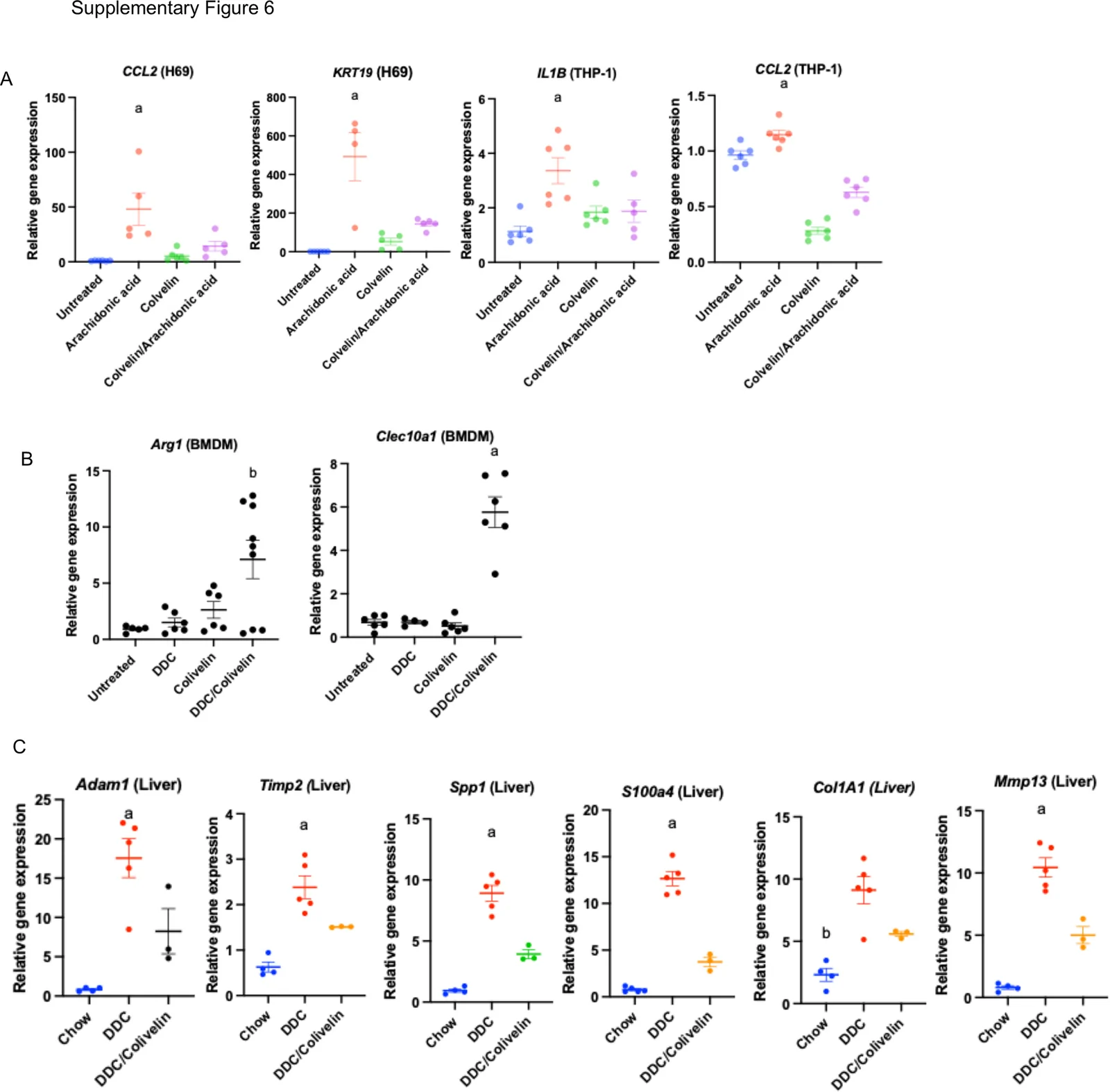


**Fig. S7:**