# Supplementary material for: Farnesoid X receptor activates STAT3 to promote fatty acid oxidation and suppress inflammatory macrophage responses in cholestasis
Source: J Biol Chem. 2026 Aug 28;302(10):113508. doi: 10.1016/j.jbc.2026.113508 (PMC13659495; doi:10.1016/j.jbc.2026.113508)
Supplement: Supplementary file 1 [file mmc1.docx]

**SUPPLEMENTARY METHODS**

**Mouse Study**

Male C57BL/6J mice were purchased from The Jackson Laboratory and kept under specific pathogen–free conditions at the University of Colorado Anschutz Medical Campus. Animals were housed in a controlled environment with consistent temperature and humidity on a 12-hour light/dark cycle, with free access to food and water. Mice were acclimated for one week before starting the experiments. All procedures followed protocols approved by the Institutional Animal Care and Use Committee (IACUC) and adhered to ARRIVE guidelines.

Only male mice were used to minimize variability associated with sex hormones. Estrogen signaling is known to influence bile acid metabolism, nuclear receptor activity, and biliary secretion, which could confound the interpretation of cholestatic injury models.

At eight weeks of age, mice were randomly assigned to experimental groups and housed separately to limit environmental variability. The treatment groups included:

- **Control (Chow):** Mice maintained on a standard laboratory diet.
- **DDC:** Mice fed a 0.1% (wt/wt) 3,5-diethoxycarbonyl-1,4-dihydrocollidine (DDC; Sigma-Aldrich) diet for 14 days to induce cholestatic liver injury.
- **DDC/GW4064 or OCA or Colivelin mice:** Mice received continuous intravenous administration of GW4064 or OCA or colivelin (GW4064; 30 mg/kg/day; OCA, 20 mg/kg/day; Colivelin; 10 mg/kg/day MedChemExpress) via central venous catheter for 14 days concurrent with the 0.1% DDC diet.
- **C-188-9 Mice:** Mice were administered the STAT3 Inhibitor C-188-9 (10 mg/kg/day; MedChemExpress) via intraperitoneal injections for 3 consecutive days.
- **Arachidonic acid (AA) Mice:** Mice received intraperitoneal injections of AA (50 mg/kg/day; Sigma-Aldrich) for three consecutive days in the presence or absence of GW4064.
- **DDC-C188-9/GW4064 Mice:** DDC-fed mice received GW4064 with or without the STAT3 inhibitor C-188-9 (10 mg/kg/day) via continuous infusion through a central venous catheter for 14 days.

Inotiv prepared custom diets in accordance with study specifications. All procedures were performed in compliance with institutional and federal guidelines for animal research.

**Tissue Collection and Sample Processing**

At the conclusion of each experimental protocol, mice were anesthetized with intraperitoneal pentobarbital prior to sample collection. Whole blood (100–400 µL) was obtained via retro-orbital sampling, allowed to clot, and centrifuged to isolate serum, which was stored at −80°C until further analysis.

Following euthanasia, liver, terminal ileum, and bone marrow were rapidly harvested. Portal venous blood was collected for metabolomic and cytokine profiling and stored at −80°C. Tissue samples were either snap-frozen in liquid nitrogen for molecular analyses or fixed in appropriate fixatives for histological and immunofluorescence studies.

Bone marrow cells were isolated from femurs and used for the generation of bone marrow-derived macrophages (BMDMs) where indicated^[1]^.

**Serum Biochemistry and Bile Acid Quantification**

Serum markers of liver injury, including alanine aminotransferase (ALT), aspartate aminotransferase (AST), alkaline phosphatase (ALP), and total bilirubin, were measured in a blinded manner by the University of Colorado Hospital Clinical Chemistry Laboratory.

Total serum bile acid concentrations were quantified using an enzymatic assay kit (Diazyme Laboratories, Cat. No. DZ042A-KY1) according to the manufacturer’s instructions. All samples were coded to ensure blinding during analysis^[2]^.

**Intrahepatic Mononuclear Cell Isolation**

IHMCs were isolated from mouse livers using an enzymatic digestion and density gradient–based protocol. Briefly, livers were perfused in situ via the portal vein with ice-cold calcium- and magnesium-free Hank’s balanced salt solution (HBSS) to remove circulating blood cells. Excised tissues were transferred to cold HBSS, finely minced, and incubated in digestion buffer containing Liberase™ R (Roche) and DNase I (Sigma-Aldrich) at 37 °C for 30 minutes with gentle agitation. Mechanical dissociation was aided by periodic pipetting.

The digested suspension was filtered through a 100 µm strainer to remove debris and centrifuged at low speed (25 × g, 3 minutes, 4 °C) to pellet hepatocytes. The supernatant containing non-parenchymal cells was collected, passed through a 70 µm filter, and centrifuged at 300 × g for 5 minutes. The resulting pellet was resuspended and layered over a 40% Histodenz gradient, followed by centrifugation at 1,500 × g for 20 minutes without braking. Mononuclear cells were collected from the interface, washed twice, and used for RNA isolation^[1]^.

**Mouse Hepatocyte Isolation**

Primary hepatocytes were isolated from freshly harvested mouse livers using a modified two-step collagenase perfusion method. Briefly, mice were anesthetized, and a 24-gauge catheter was inserted into the inferior vena cava to enable in situ liver perfusion. The liver was sequentially perfused at 37 °C with pre-warmed solutions as follows: (i) Earle’s Balanced Salt Solution (EBSS) supplemented with EGTA (Thermo Fisher Scientific; Cat. No. 14155) for 15 minutes to chelate calcium and disrupt intercellular junctions; (ii) liver perfusion medium (Thermo Fisher Scientific; Cat. No. 17701) for 6 minutes; and (iii) liver digest medium containing collagenase (Thermo Fisher Scientific; Cat. No. 17703) for 15 minutes to facilitate enzymatic dissociation. Following digestion, the liver was excised, gently dispersed, and filtered through a 70-µm cell strainer to obtain a single-cell suspension. Hepatocytes were washed in William’s E medium (Thermo Fisher Scientific; Cat. No. 32551) and purified by low-speed centrifugation (25 × g, 5 minutes) to selectively pellet viable hepatocytes. The isolated hepatocyte fraction was used for downstream applications, including RNA and protein extraction^[3]^.

## **siRNA-Mediated STAT3 Knockdown and Treatment**

Murine macrophage Raw 264.7 and human hepatoma Huh7 cells (ATCC) were cultured under standard conditions and subjected to siRNA-mediated gene silencing. Cells were transfected with STAT3-specific siRNA (siRNA-STAT3, Cat. No. L-003544-00-0005; Horizon Discovery) or a non-targeting control siRNA (siRNA-NTC; Cat. No. D-001810-10-05; Horizon Discovery using DharmaFECT transfection reagent according to the manufacturer’s instructions. Transfections were performed for 24 hours in serum-containing DMEM. Following transfection, cells were treated with arachidonic acid (AA; 50 µM) in the presence or absence of the FXR agonist GW4064 (5 µM) overnight. Treatments were carried out in DMEM supplemented with serum and transfection reagents as indicated. In a separate set of experiments, cells were processed under identical transfection conditions and subjected to additional treatments as specified in the corresponding figure legends^[4]^.

## **Immunoblot Analysis**

Total protein was extracted from liver or cultured cells using M-PER™ or T-PER™ or NE-PER lysis buffers (Thermo Fisher Scientific) according to the manufacturer’s instructions. Protein concentrations were determined using a standard protein BCA quantification assay. Equal amounts of protein were separated by SDS polyacrylamide gel electrophoresis using 4–20% gradient gels (Bio-Rad Laboratories) and subsequently transferred onto polyvinylidene difluoride (PVDF) membranes (MilliporeSigma). Membranes were blocked with the appropriate 5% nonfat milk and incubated overnight at 4 °C with primary antibodies. Following primary incubation, membranes were washed and incubated with horseradish peroxidase (HRP) conjugated secondary antibodies. Protein bands were detected using enhanced chemiluminescence (ECL) reagents and visualized using an imaging system. A detailed list of primary and secondary antibodies, including dilution and source information, is provided in **Supplementary Table 2**.

**Histological Analysis**

Following euthanasia, the liver was removed and a small tissue section was placed in a histology cassette (Cancer Diagnostics, cat. no. STG1000) containing a biopsy foam pad (Simport Inc., cat. no. M476-1). The cassette was immersed in 10% neutral buffered formalin phosphate (Fisher Scientific, cat. no. SF100-4) for 24 h. The following day, the cassette was transferred to 70% ethanol (Decon Laboratories Inc., cat. no. DSP-MD43) for 1 h to remove residual formalin. It was then placed in a clean container containing fresh 70% ethanol and transferred to the institutional histology core facility for further tissue processing, paraffin embedding, sectioning, and staining. Liver tissues were paraffin-embedded and sectioned at a thickness of 4 µm. Sections were stained with hematoxylin and eosin (H&E) using standard procedures to evaluate tissue morphology. For immunohistochemistry, paraffin sections underwent heat-induced antigen retrieval in 10 mM citrate buffer containing Tween-20 (pH 6.0) for 10 min at 110°C at NxGen, P.C. Sections were incubated with primary antibodies against F4/80, a macrophage marker (Cell Signaling Technology, cat. no. 70076), and cytokeratin 19 (CK19), a biliary epithelial marker (Proteintech, cat. no. 14965-1-AP). Antibody detection was performed using a horseradish peroxidase-based chromogenic system on an automated Ventana/Roche staining platform. Sections were counterstained with hematoxylin, dehydrated, cleared, and mounted.All histological staining, immunohistochemical processing were performed through the University of Colorado Cancer Center Histology Core. Paraffin-embedded liver sections (4-5 µm) were deparaffinized, rehydrated through graded alcohols, and stained with Weigert’s iron hematoxylin for 8 min, followed by washing in running tap water. Sections were then stained with Picro-Sirius Red solution (Direct Red 80 in saturated picric acid) for 60 min, washed in acidified water, rapidly dehydrated through 100% ethanol, cleared in xylene, and coverslipped. Collagen deposition was evaluated by polarized-light microscopy.

For each CK19-stained liver section, 3-4 representative, non-overlapping images were acquired at 100× magnification using an Olympus BX51 microscope equipped with an Olympus DP73 digital camera and CellSens software. Identical acquisition settings were used for all samples. TIFF images were analyzed using SlideBook software (Intelligent Imaging Innovations, Denver, CO). After segmentation of the tissue area, positive pixels were quantified and expressed as a percentage of total tissue pixels. Values from the 3–4 images were averaged to obtain one value per animal^[1]^.

**IL-6 Enzyme-Linked Immunosorbent Assay (ELISA)**

Interleukin-6 (IL-6) levels were quantified in the mouse serum IL-6. Mouse IL-6 ELISA Kit (Cat. No. BMS603-2; Thermo Fisher Scientific). Assays were performed according to the manufacturer’s protocols. Briefly, samples and standards were added to pre-coated microplate wells and incubated to allow IL-6 binding. Following washing steps, detection antibodies and enzyme conjugates were applied, and the signal was developed using a chromogenic substrate. Absorbance was measured at the recommended wavelength using a microplate reader. IL-6 concentrations were calculated from standard curves generated using recombinant IL-6 standards and expressed as pg/mL All samples were analyzed in duplicate or triplicate.

## **Caspase-8 and Caspase-3/7 Activity Assay**

Caspase-8 and Caspase-3/7 activity was measured using the Caspase-Glo® 8 and Caspase-Glo® 3/7 Assay System ( Cat No: G8200; Cat. No. G8090; Promega) according to the manufacturer’s instructions. Briefly, cells were plated in white-walled 96-well plates and subjected to the indicated treatments. An equal volume of Caspase-Glo® 8 and Caspase-Glo® 3/7 reagent was added directly to each well, followed by gentle mixing to induce cell lysis and initiate the luminescent reaction. Samples were incubated at room temperature for 30–60 minutes to allow cleavage of the luminogenic caspase-3/7 substrate. Luminescence, proportional to caspase activity, was measured using a plate reader. Background signals from media-only wells were subtracted, and data were normalized to control conditions.

**Immunofluorescence Staining**

BMDM and Raw 264.7cells were fixed with 4% paraformaldehyde. After permeabilization with Triton X-100, sections were blocked with serum-containing buffer to reduce nonspecific binding. Fc receptor blocking was included where necessary. Sections were incubated overnight at 4 °C with primary antibodies against Acetylated STAT3, P300, and pSTAT3, followed by incubation with fluorophore-conjugated secondary antibodies. Nuclei were counterstained with DAPI-containing mounting medium. Images were captured using confocal microscopy under identical acquisition settings across all groups to ensure comparability. The confocal microscopy was done in Zeiss LSM780 spectral microscope at the University of Colorado Denver Anschutz campus at the Advanced Light Microscopy core.

**RNA Isolation and Quantitative PCR**

Total RNA was extracted from liver tissue, isolated cells, or cultured macrophages using TRIzol reagent (Invitrogen) or the RNeasy Mini Kit (QIAGEN) according to manufacturer protocols. RNA was reverse transcribed using the iScript™ cDNA Synthesis Kit (Bio-Rad). Quantitative real-time PCR was performed using TaqMan assays (Thermo Fisher Scientific) with appropriate gene-specific probes. Reactions were run in duplicate or triplicate, and gene expression was normalized to internal controls. Relative gene expression was calculated using the 2^−ΔΔCt method. Target gene Ct values were normalized to the corresponding housekeeping gene, and the mean ΔCt of the designated control group was used as the calibrator for each experiment. Relative expression values therefore represent fold change relative to the appropriate control group, with individual biological replicates allowed to vary around the normalized group mean.

**RNA Sequencing and Bioinformatic Analysis**

RNA extracted from liver was used for library preparation with the Illumina TruSeq platform and sequenced using an Illumina system in the University of Colorado Anschutz Genomics Core. Raw reads were processed to remove low-quality sequences, adapters, and contaminants. Clean reads were aligned to the mouse reference genome (GRCm39) using HISAT2. Transcript quantification was performed using StringTie, and gene expression levels were calculated as fragments per kilobase per million reads (FPKM). Differential expression analysis was conducted using DESeq2, applying a threshold of false discovery rate (FDR) < 0.05 and fold change ≥ 2. **LC Sciences (Houston, TX, USA) performed the bioinformatics analysis** on the raw RNA sequencing data using their standard processing pipeline Functional enrichment was evaluated using Gene Set Enrichment Analysis (GSEA). Sample relationships were assessed using principal component analysis and Pearson correlation. Variant analysis was performed with SAMtools and annotated using ANNOVAR. RNA-seq datasets have been deposited in the NCBI Gene Expression Omnibus under accession numbers PRJNA1454983 and PRJNA1401481.

## **Co-culture Experiments**

Newly purchased cell lines (ATCC, Manassas, Virginia, USA) were cultured. Human cholangiocyte H69 cells (H69) were maintained in DMEM/Ham’s F-12 (1:1) medium supplemented with 10% defined fetal bovine serum, penicillin (100 U/mL), streptomycin (100 µg/mL), adenine (24 µg/mL), insulin (5 µg/mL), epinephrine (0.2 µg/mL), triiodothyronine (2 nM), transferrin (5 µg/mL), epidermal growth factor (10 ng/mL), and hydrocortisone (0.5 µg/mL). Cells were cultured at 37 °C in a humidified atmosphere containing 5% CO₂. Culture medium was replaced every 2–3 days, and cells were passaged at 80–85% confluence using 0.05% trypsin–EDTA. Only low-passage cells (passage <25) were used for experiments. For transwell co-culture assays, H69 cells were seeded onto permeable inserts (cat no 140652**;** Thermo Fisher Scientific), while THP-1 monocytes were plated in the lower chamber of 12-well plates (Thermo Fisher Scientific). This setup allowed paracrine interactions without direct cell-cell contact. H69 cells were treated with vehicle control or AA (50 µM) overnight before analysis. Following co-culture, RNA was isolated separately from each cell population for further analysis.

**NADH Measurement:**

NADH levels in liver homogenate were measured using a commercial NADH Assay Kit (AAT Bioquest, Cat. No. 15273, Pleasanton, CA) according to the manufacturer’s instructions. Absorbance was measured at 460 nm using a microplate spectrophotometer, and values were normalized according to the assay guidelines.

**Acetyl-CoA Measurement:**

Acetyl-CoA levels in liver homogenate were quantified using a commercial Acetyl-CoA Assay Kit (Novus Biologicals, Cat. No. NBP3-24455, Centennial, CO) according to the manufacturer’s instructions. Absorbance was measured at 340 nm using a microplate spectrophotometer, and values were normalized according to the assay guidelines.

## **Metabolomics and Oxylipin Analysis**

Polar metabolites and oxylipins were extracted from homogenized mouse liver tissue or serum using a cold solvent system consisting of methanol:acetonitrile:water (5:3:2, v/v/v). Tissue samples were processed at a ratio of 15 mg per mL of extraction solvent. Samples were homogenized using glass bead disruption for 5 minutes, followed by vigorous vortexing for 30 minutes at 4 °C to ensure efficient metabolite extraction. The homogenates were then centrifuged at 18,213 × g for 10 minutes at 4 °C, and the clarified supernatants were collected for analysis. Metabolomic profiling was performed using ultra-high-performance liquid chromatography coupled to high-resolution mass spectrometry (UHPLC-HRMS) on a Vanquish UHPLC system interfaced with an Exploris mass spectrometer (Thermo Fisher Scientific). Samples were injected at a volume of 10 µL for all analyses.For global metabolomics and oxylipin profiling, metabolites were separated using a 1.7 µm, 2.1 × 150 mm Kinetex C18 column under a rapid gradient elution method. Targeted bile acid profiling was performed on the same extracts using a 1.7 µm, 2.1 × 100 mm Acquity BEH C18 column with a modified gradient optimized for bile acid separation. Raw mass spectrometry data were converted to mzXML format using RawConverter and processed for metabolite identification and quantification. Metabolite annotation was based on accurate mass measurements, isotopic distribution patterns (including ¹³C signatures), and retention time matching against the KEGG database, complemented by an in-house library of authenticated standards. Peak detection and integration were performed using Maven. Quality control was ensured using pooled technical replicates, which were analyzed at the beginning, midpoint, and end of each analytical batch to monitor instrument performance and data reproducibility.

**Chromatin Immunoprecipitation (ChIP) Assay**

Chromatin immunoprecipitation (ChIP) was performed using isolated primary mouse hepatocytes or liver tissue homogenates with the EZ-ChIP™/Magna ChIP™ G Kit (MilliporeSigma) in accordance with the manufacturer’s instructions. Briefly, samples were crosslinked with formaldehyde to stabilize protein–DNA interactions, followed by glycine-mediated quenching. Nuclei were isolated, and chromatin was sheared by sonication to generate fragments of appropriate size. The fragmented chromatin was incubated with antibodies against FXR to assess binding at bile acid-regulated promoter regions (*Stat3*) or STAT3 to evaluate occupancy at the *Cpt1a and Cpt1b* promoters. Species-matched IgG controls were included to account for nonspecific binding. After immunoprecipitation, crosslinks were reversed, and DNA was purified for downstream analysis. Enrichment of target genomic regions was quantified by PCR using gene-specific primers. ChIP signals were normalized to input chromatin and expressed relative to IgG controls.

**Annexin V Apoptosis Analysis by Flow Cytometry**

Apoptosis in primary mouse hepatocytes or Huh7 cells was quantified using an Annexin V-based flow cytometry assay. Hepatocytes were isolated and subjected to indicated experimental treatments, then harvested and washed twice with cold phosphate-buffered saline (PBS). Cells were resuspended in 1× Annexin V binding buffer at a concentration of ~1 × 10⁶ cells/m. Cells were stained with Annexin V conjugated to phycoerythrin (PE) (Cat No: 88-8102-72 Thermofisher Scientific,Waltham, MA) according to the manufacturer’s protocol. To distinguish late apoptotic and necrotic cells, viabilty staining solution was added immediately before acquisition. Samples were incubated for 10–15 minutes at room temperature in the dark. Compensation and spectral unmixing were performed using single-stained controls and unstained controls. Data acquisition was performed at the University of Colorado Cancer Center Flow Cytometry Core Facility using a flow cytometer (Agilent Novocyte Penteon). Appropriate controls, including unstained and single-stained samples, were used for spectral unmixing and compensation.

**CD95-Flow Cytometry Analysis**

Primary mouse hepatocytes were isolated from liver tissue using established protocols ^[4]^. Cells were washed, pelleted, and resuspended in staining buffer (Thermo Fisher Scientific) before antibody labeling. Cells were incubated with fluorophore-conjugated antibodies against apoptotic CD95 (Fas receptor, cat No; 17-0951-8, ThermoFisher Scientific) according to standard staining procedures. After incubation, cells were washed to remove excess antibodies and prepared for flow cytometric acquisition. Data acquisition was performed at the University of Colorado Cancer Center Flow Cytometry Core Facility using a spectral flow cytometer (e.g., Bio-Rad YETI; Bio-Rad Laboratories). Appropriate controls, including unstained and single-stained samples, were used for spectral unmixing and compensation. Flow cytometry data were analyzed using Kaluza (Beckman Coulter). Cell populations were identified based on forward- and side-scatter properties and marker expression.

**Statistical Analysis**

Quantitative real-time PCR (qRT–PCR) assays were performed in technical triplicates for each biological replicate. Mean values were used to calculate group averages, which are presented as mean ± standard error of the mean (SEM). Animal experiments were typically conducted with group sizes ranging from *n* = 3–5 per group, with exact sample sizes specified in individual figure legends. For in vitro studies, experiments were performed with at least three independent replicates unless otherwise indicated. Statistical analyses were carried out using GraphPad Prism and R. Comparisons among multiple groups were evaluated using one-way analysis of variance (ANOVA) followed by Tukey’s post hoc test to correct for multiple comparisons. Pairwise comparisons between two groups were performed using unpaired, two-tailed Student’s *t*-tests. Analyses involving FXR agonists were conducted independently for each treatment condition where appropriate. A *P* value < 0.05 was considered statistically significant. All samples were coded, and data acquisition and analysis were performed in a blinded manner to minimize bias. Data are presented as mean ± SEM to indicate the precision of the estimated group mean. Individual biological replicate values are shown where possible, and sample sizes are specified in the corresponding figure legends. Statistical significance was defined using the indicated tests.

**LEGENDS FOR SUPPLEMENTARY FIGURES**

**Supplementary Figure 1. Transcriptomic analysis of hepatic gene expression during DDC-induced cholestasis and FXR agonist treatment.**

(A) Volcano plots illustrating liver DEGs comparing DDC vs Chow, DDC/GW4064 vs DDC, and DDC/OCA vs DDC mice. Red dots represent significantly upregulated genes, while blue dots signify significantly downregulated genes (FDR < 0.05, |log₂FC| ≥ 1).

(B) Venn diagram illustrating overlap among significant DEGs identified in DDC vs Chow, DDC/GW4064 vs DDC, and DDC/OCA vs DDC mice. Shared gene sets indicate common transcriptional programs regulated during cholestatic injury and following FXR activation.

**(C)** Heatmap showing the expression of selected STAT3- and fatty acid oxidation (FAO)-associated genes (Socs3, Stat3, Socs1, Acox1, and Cpt1a) across liver RNA-seq samples from Chow, DDC, DDC/GW4064, and DDC/OCA groups (n = 3 mice per group).

**Supplementary Figure 2. Metabolomic profiling of arachidonic acid–derived lipid mediators during cholestatic injury and FXR activation.**

(A) Principal component analysis (PCA) showing separation of metabolomic profiles among Chow, DDC, and DDC/FXR agonist–treated groups.

(B–D) Pairwise AA metabolism pathway analysis of metabolomics data comparing Chow vs. DDC, DDC vs. DDC/GW4064, and DDC vs. OCA mice (E) heatmap showing differential abundance in DDC and DDC/GW4064 mouse livers of oxylipin metabolites and lipid mediators identified by metabolomic analysis. Heat map analysis showed increased expression of several STAT3-associated cytochrome P450 genes in DDC/GW4064 mice compared with Chow and DDC mice.

(F) PGD2 overnight incubation with cultured Raw 264.7cells induces macrophage anti-inflammatory gene expression.

Statistical analysis was performed by 2 tailed unpaired Student’s t-test. a = p<0.05 vs. all other groups. (n = 3-5 mice per group); Three independent biological experiments for in vitro cell study.

**Supplementary Figure 3. Transcriptomic and functional analysis of cell death-associated genes and inflammatory signaling pathways during cholestatic injury.**

(A-C) Heatmap of RNA-seq expression of genes involved in apoptotic genes showing clustering of differentially expressed genes in pairwise comparison (A) DDC and Chow) (B) DDC and DDC/GW4064 (C) DDC and DDC/OCA mouse livers.

(D-F) Heatmap of RNA-seq expression of genes associated with anti-inflammatory genes in DDC mouse liver and (D) Chow, (E) DDC/GW4064 (F) DDC/OCA.

(G) Quantitative PCR analysis of anti-inflammatory macrophage genes in lHMCs isolated from liver of the four experimental group. Expression of anti-inflammatory macrophage markers, *Cd163, Retnlg,* and *Chil3* are upregulated in GW4064 and OCA treated mice.

Data in (G) is presented as mean ± SEM. Statistical significance was determined using one-way ANOVA with appropriate multiple-comparison correction. a = p<0.05 vs. all other groups. (n = 3-5 mice per group);

**Supplementary Figure 4. FXR activation restores bile acid profiles during cholestatic injury.**

(A) Principal component analysis of metabolomics data illustrating separation of bile acid profiles among Chow vs. DDC and DDC vs. DDC/GW4064 mice.

(B, C) Heatmap and fold change analysis of liver bile acid metabolites showing enrichment of primary bile acids in DDC mice compared to Chow mice, which was reversed in DDC mice treated with GW4064. (n = 3-5 mice per group); Three independent biological experiments for in vitro cell study.

**Supplementary Figure 5. Effects of STAT3 inhibition on liver injury, macrophage activation, and inflammatory signaling.**

(A) Serum biochemical markers of liver injury in Chow and STAT3 inhibitor (C188-9) treated mice.

(B) Immunohistochemistry for macrophage marker F4/80 and cholangiocyte marker CK19 following STAT3 inhibition with C-188-9 in Chow mice, showing increased macrophage infiltration and ductular response following STAT3 inhibition.

(C) Gene expression analysis in macrophages isolated from mouse liver following STAT3 inhibition by i.p. injection of C188-9, demonstrating increased pro-inflammatory macrophage gene expression with STAT3 inhibition.

(D) Gene expression analysis in human cholangiocyte cell line (H69 cells; upper wells) treated overnight with AA with and without STAT3 inhibitor (C188-9) and co-cultured with THP-1 cells (lower wells). Expression of *KRT19, CCL2,* and *IL1B* in H69 cells exposed to AA is enhanced following STAT3 inhibition, while *CCL2, IL1B* and *CXCL1* expression are similarly upregulated in the THP-1 cells with STAT3 inhibition.

Data are presented as mean ± SEM (n = 3-4 mice per group; or 3 biological replicates for cell culture experiments). Statistical significance was determined using one-way ANOVA with appropriate multiple-comparison correction. a = p<0.05 vs. all other groups; d = p<0.05 vs. AA. (n = 3-5 mice per group); Three independent biological experiments for in vitro cell study.

**Supplementary Figure 6. STAT3 activation suppresses arachidonic acid–induced inflammatory signaling in hepatocytes and macrophages.**

(A) Gene expression analysis of inflammatory mediators (*CCL2, KRT19, IL1B, CCL2,*) in cholangiocyte (H69) cell line co-cultured with human macrophage (THP-1) cells treated with AA and/or the STAT3 activator colivelin.

(B*)* Gene expression analysis by qPCR of cultured BMDMs incubated overnight with the indicated treatments, demonstrating upregulated *Arg1* and *Clec10a1* expression with combined DDC and colivelin treatment.

(C) qPCR analysis of fibrosis-related genes from liver in experimental groups, showing upregulation in DDC mice, which was suppressed in DDC mice treated with colivelin (STAT3 activator).

Data are presented as mean ± SEM. (n = 3-5 mice per group); Statistical significance was determined using one-way ANOVA with appropriate multiple-comparison correction. a = p<0.05 vs. all other groups; b = p<0.05 vs. DDC. (n = 3-5 mice per group); Three independent biological experiments for in vitro cell study.

**Supplementary Figure 7. Alterations of STAT3-dependent regulation of fatty acid oxidation pathways during cholestatic liver injury.**

(A) Heatmap of RNA-seq expression data of genes involved in fatty acid oxidation shows downregulation of the majority of genes in DDC (cholestatic) vs. Chow mice. (B) Pathway analysis of RNA-seq expression data of mouse liver shows that STAT3-regulated FAO-metabolism genes are altered in DDC mice and primarily upregulated in GW4064-treated DDC mice. (C) Immunoblot analysis of P300 protein expression in cultured Raw 264.7 macrophages following incubation overnight incubation with acetyl-CoA.(D-E) Confocal microscopy of cultured Raw 264.7 macrophages shows co-localization (blue arrow) of P300 with mitotracker that is induced by incubation with Acetyl-CoA (upper panel), and concurrent co-localization (blue arrow) of acetyl-STAT3 within mitotracker (lower panel). Representative images of 3 biological replicates are shown.Statistical analysis was performed by 2 tailed unpaired Student’s t-test. a = p<0.05 vs. all other groups. (n = 3-5 mice per group); Three independent biological experiments for in vitro cell studies.

**Supplementary Table 1. List of TaqMan Probes**

**Mouse**

| Gene | Probe Set |
| --- | --- |
| *Arg1* | [Mm00475988](https://www.thermofisher.com/taqman-gene-expression/product/Mm00475988_m1?CID=&ICID=&subtype=) |
| *Cpt1a* | Mm01231183 |
| *Cpt2* | Mm00487205 |
| *Klf4* | Mm00516104 |
| *Retnlg* | Mm00516104 |
| *Ccl2* | Mm00441242 |
| *Cd68* | Mm03047343 |
| *Cd80* | Mm00711660 |
| *Chil3* | Mm00657889 |
| *Slc10a2* | Mm00488258 |
| *Myc* | Mm00487804 |
| *Hmox1* | Mm00516005 |
| *Il-1b* | Mm00434228 |
| *Maf* | Mm01200905 |
| *Cd163* | Mm00474091 |
| *Itgam* | Mm00434455 |
| *Stat3* | Mm01219775 |
| *Col1A* | Mm00802300 |
| *Spp1* | Mm00436767 |
| *S100a4* | Mm00803371 |
| *Mmp13* | Mm00439491 |
| *Cxcl1* | Mm04207460 |
| *Adgre1* | Mm00802529 |
| *Cpt1b* | Mm00487200 |
| *Aco2* | Mm00475658 |
| *Hadhb* | Mm01210656 |
| *Acox1* | Mm01246834 |

**Human**

| Gene | Probe Set |
| --- | --- |
| *ABCB11* | Hs00994811 |
| *ABCG8* | Hs00223690 |
| *STAT3* | Hs00374280 |
| *ABCC2* | Hs00960489 |
| *NR0B2* | Hs00222677 |
| *KRT19* | Hs00174128 |
| *IL1B* | Hs01555410 |
| *CCL2* | Hs00234140 |
| *CXCL1* | Hs00236937 |

**Supplementary Table 2. List of Antibodies**

| **Antibody** | **Catalog No** | **Dilution** | **Vendor** |
| --- | --- | --- | --- |
| Acetyl-Stat3 | A13982  ES8435 | 1:1000 | Thermofisher Scientific/ELK biotech |
| STAT3 | 8690 | 1:1000 | Cell Signaling |
| Phospho STAT3 | 9145 | 1:1000 | Cell Signaling |
| P300 | 57625 | 1:1000 | Cell Signaling |
| GRB2 | 3972 | 1:1000 | Cell Signaling |
| Anti-Mouse- IgG-HRP | HAF007 | 1:5000 | R&D systems |
| Anti-Rabbit -IgG-HRP | 3972 | 1:5000 | Santa Cruz Biotechnology |

1	Ghosh S, Devereaux MW, Anderson AL, Anderson CC, D'Alessandro A, Orlicky DJ, Sokol RJ. Dietary Lauric Acid Suppresses Inflammation, Cholestasis, Hepatocyte Injury, and Senescence in 3,5-Diethoxycarbonyl-1,4-Dihydrocollidine-induced Inflammatory Cholangiopathy. *Cell Mol Gastroenterol Hepatol* 2026; **20**(5): 101731 [PMID: 41571094 PMCID: PMC12990384 DOI: 10.1016/j.jcmgh.2026.101731]

2	Ghosh S, Devereaux MW, Anderson AL, Gehrke S, Reisz JA, D'Alessandro A, Orlicky DJ, Lovell M, El Kasmi KC, Shearn CT, Sokol RJ. NF-kappaB Regulation of LRH-1 and ABCG5/8 Potentiates Phytosterol Role in the Pathogenesis of Parenteral Nutrition-Associated Cholestasis. *Hepatology* 2021 [PMID: 34310734 DOI: 10.1002/hep.32071]

3	Lu D, Liu Y, Luo Y, Zhao J, Feng C, Xue L, Xu J, Wang Q, Yan T, Xiao P, Krausz KW, Gonzalez FJ, Xie C. Intestinal farnesoid X receptor signaling controls hepatic fatty acid oxidation. *Biochim Biophys Acta Mol Cell Biol Lipids* 2022; **1867**(2): 159089 [PMID: 34856412 PMCID: PMC8864892 DOI: 10.1016/j.bbalip.2021.159089]

4	Ghosh S, Devereaux MW, Anderson AL, El Kasmi KC, Sokol RJ. Stat3 role in the protective effect of FXR Agonist in parenteral nutrition-associated cholestasis. *Hepatol Commun* 2023; **7**(3): e0056 [PMID: 36848082 PMCID: PMC9974070 authors have nothing to report. DOI: 10.1097/HC9.0000000000000056]
